# Magnetically Controlled Reversible Photomagnetic Nanoactuators for Dynamic Contrast Enhancement in Optical Coherence Tomography

**DOI:** 10.1002/adma.202419235

**Published:** 2025-06-19

**Authors:** Myeongsoo Kim, Samuel M. A. Morais, Anamik Jhunjhunwala, Shivashankar Subramanian, Paul S. Pelkowski, Stanislav Y. Emelianov

**Affiliations:** ^1^ Wallace H. Coulter Department of Biomedical Engineering Georgia Institute of Technology and Emory University School of Medicine Atlanta GA 30332 USA; ^2^ Petit Institute for Bioengineering and Biosciences Georgia Institute of Technology Atlanta GA 30332 USA; ^3^ School of Electrical and Computer Engineering Georgia Institute of Technology Atlanta GA 30332 USA

**Keywords:** dynamic contrast enhancement, magnetic control, optical coherence tomography, photomagnetic nanoactuator, reversible signal modulation

## Abstract

Optical coherence tomography (OCT) is a high‐resolution imaging modality that detects scattered optical signals from light–tissue interaction. However, the ability of OCT to visualize specific molecular and cellular events is hindered by the lack of effective exogenous contrast agents capable of producing noticeable imaging contrast. Here, photomagnetic nanoactuators with ≈90% scattering efficiency in the second near‐infrared window, capable of reversible magnetic field‐mediated modulation of optical scattering and, therefore, OCT signal, are proposed. These nanoactuators consist of a magnetite core and gold shell, functioning as a reversible magnetic actuator and an optical scatterer, respectively. When exposed to the external magnetic field, photomagnetic nanoactuators are assembled into chain structures via magnetostatic interactions between nearby nanoactuators, reducing their optical scattering and, consequently, the OCT signal. Upon removal of the magnetic field, the nanoactuators are disassembled, restoring their scattering property and OCT signal. It is demonstrated that this magnetically controlled OCT response allows enhanced particle‐associated signal detection with suppressed background signal via image subtraction before and after magnetic field treatment, both in vitro and in vivo. The developed nanoactuator platform offers a strategy for dynamic enhancement of OCT contrast, potentially broadening the utility of OCT for noninvasive cell tracking and molecular diagnostic imaging.

## Graphical abstract

Photomagnetic nanoactuators with ≈90% scattering efficiency in the second near‐infrared window are demonstrated to exhibit magnetic field‐controlled modulation of optical scattering and optical coherence tomography signals. These nanoactuators are reversibly assembled and disassembled under the external magnetic field modulation, enabling enhanced particle‐associated signal detection with suppressed background signal both in vitro and in vivo.

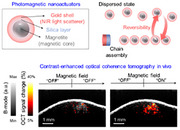

## 1 Introduction

Optical coherence tomography (OCT) is an imaging modality that provides millimeter‐scale depth penetration and submicrometer spatial resolution, enabling the generation of microscale cross‐sectional images of biological tissues.^[^ ^1^, ^2^, ^3^, ^4^ ^]^ OCT is conceptually similar to ultrasound B‐mode imaging or radar, but uses a near‐infrared (NIR) light source for image reconstruction.^[^ ^3^, ^4^ ^]^ As an optical imaging technique, OCT thus achieves spatial resolution an order of magnitude higher than conventional ultrasound imaging.^[^ ^4^ ^]^ Specifically, OCT captures depth‐resolved backscattered light resulting from interactions between incident light and tissue molecules.^[^ ^1^, ^2^, ^3^, ^4^ ^]^ Because biological tissues exhibit heterogeneity in optical refractive indices, OCT enables imaging of fine tissue microstructures without requiring tissue excision.^[^ ^1^, ^4^ ^]^ Therefore, since its invention, OCT has been widely adopted for various biomedical applications, such as cancer diagnosis,^[^ ^5^, ^6^ ^]^ ophthalmology,^[^ ^7^, ^8^ ^]^ surgical guidance,^[^ ^9^ ^]^ cardiology,^[^ ^10^, ^11^ ^]^ and pulmonology.^[^ ^12^ ^]^

While endogenous molecules, such as cell nuclei, mitochondria, and hemoglobin, are typically used to characterize tissue morphology with OCT,^[^ ^13^, ^14^, ^15^, ^16^ ^]^ exogenous contrast agents with large scattering cross‐sections at NIR wavelengths, such as gold nanoparticles,^[^ ^17^, ^18^, ^19^, ^20^ ^]^ copper sulfide quantum dots,^[^ ^21^ ^]^ and microbubbles,^[^ ^22^ ^]^ can enhance imaging by providing better visualization of specific molecular and cellular events with enhanced contrast. Despite robust scattering responses from designed contrast agents, they often encounter challenges in clearly identifying specific biomolecular processes in tissue due to poor efficiency or difficulty in coupling the agents to the targeted biological processes, leading to unnoticeable signals compared to the background.

To address these challenges, designing OCT contrast agents that respond to external stimuli, such as heat,^[^ ^23^, ^24^, ^25^ ^]^ ultrasound,^[^ ^26^ ^]^ or magnetic field,^[^ ^27^, ^28^, ^29^ ^]^ to modulate their optical scattering responses can enhance the signal detection of such agents by comparing OCT signals between pre‐ and post‐stimulus treatment via image subtraction.^[^ ^23^, ^24^, ^25^, ^26^, ^27^, ^28^, ^29^, ^30^ ^]^ When the signal changes from the stimulus‐responsive contrast agents pre‐ and post‐stimulus treatment is significantly greater than that of background tissue, this approach can provide enhanced OCT contrast highlighting the regions where the agents are located. Across various external stimuli, the utilization of magnetic fields, in particular, offers distinct advantages since it provides spatiotemporal control with minimal tissue interference. For instance, magnetomotive OCT with the use of magnetic nanoparticles, such as magnetite (Fe_3_O_4_) nanospheres,^[^ ^6^, ^27^ ^]^ has been employed as a tool to enhance imaging contrast by causing mechanical perturbation in surrounding tissue through lateral movement or rotation of the magnetic nanoparticles when exposed to an external magnetic field.^[^ ^27^, ^28^, ^31^ ^]^ However, the performance of this technique primarily depends on tissue stiffness and elasticity to produce measurable imaging signals and contrast,^[^ ^28^, ^32^ ^]^ posing challenges in characterizing soft or fluid‐like biological tissue specimens, such as blood.^[^ ^28^ ^]^ Furthermore, significant image contrast from mechanically perturbed tissue regions could require either a large accumulation or substantial displacement of magnetic nanoparticles within the target tissue,^[^ ^31^ ^]^ as biological tissue also exhibits fluctuations. Additionally, the use of magnetic nanoplatelets has been shown to control optical absorption and, consequently, backscattering from tissue by magnetically modulating particle orientation.^[^ ^30^ ^]^ However, in this system, the changes in OCT signals are highly dependent on the angle between the light and the particle orientation. The effectiveness of signal changes may be diminished in vivo due to random scattering of the incident light by endogenous scatterers, such as blood. Furthermore, magnetic nanoparticles alone do not exhibit strong scattering responses at NIR wavelengths due to their low scattering cross‐section, which limits the ability to monitor particles without the application of an external magnetic field.

**Scheme 1:**
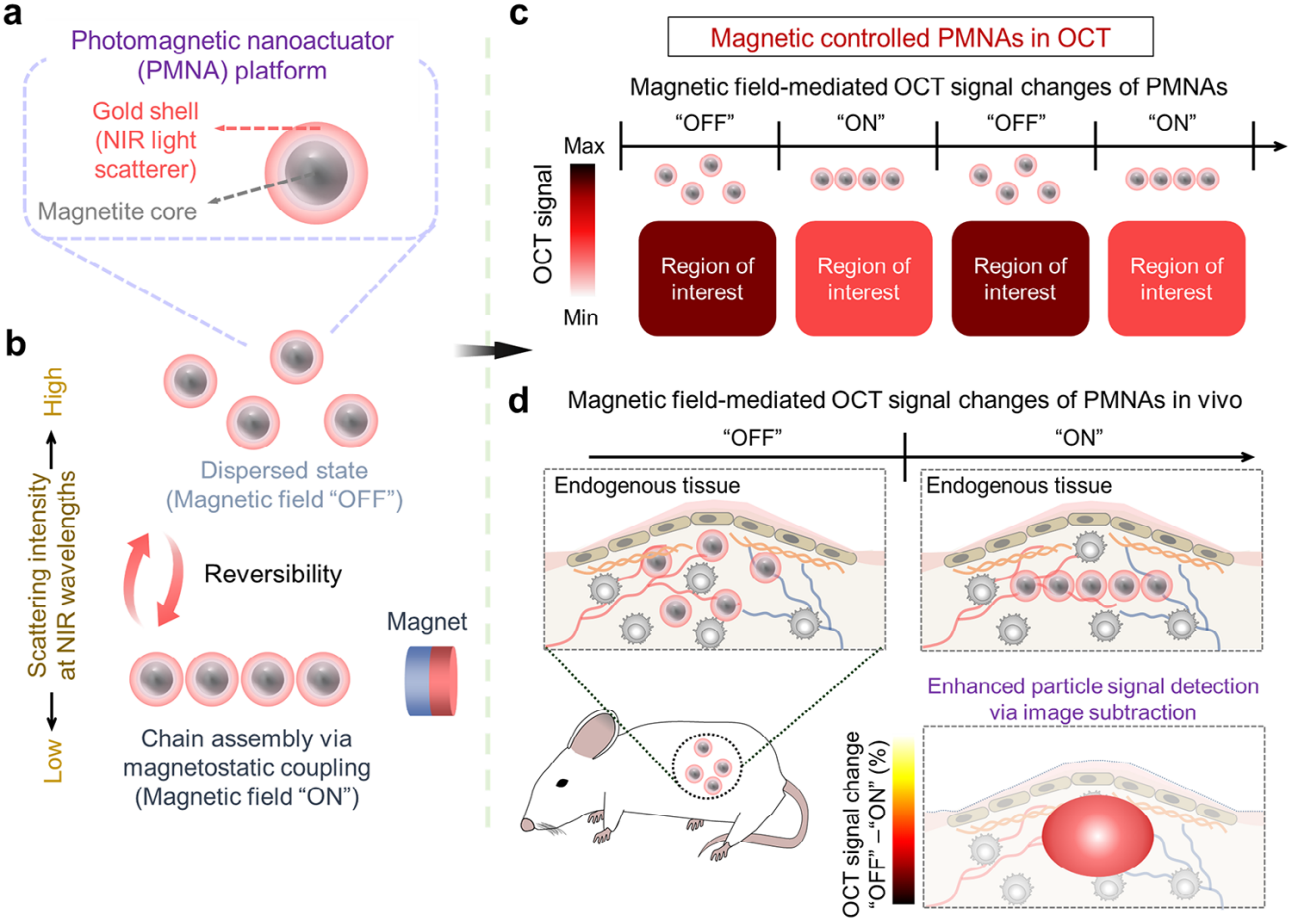
Schematic summary of the experimental approach used in this study. a) PMNAs consist of a magnetite core and a gold shell, serving as a magnetic core and an optical scatterer, respectively. b) When exposed to an external magnetic field, PMNAs assemble into chain‐like structures through magnetostatic coupling between neighboring particles, resulting in reduced scattering in the second NIR window. Upon removal of the magnetic field, the PMNAs disassemble into a monodisperse state, restoring their scattering intensity. c,d) This magnetically controllable optical scattering property enables PMNAs to modulate OCT signals (c), allowing enhanced particle‐associated signal detection with suppressed background signal through pixel‐by‐pixel subtraction of images before and after magnetic field application in both in vitro and in vivo settings (d).

Herein, we introduce a spherical photomagnetic nanoactuator (PMNA) that is composed of iron oxide core and gold shell as a material platform to enhance particle‐associated signal detection with suppressed background signal in OCT under the control of an external magnetic field (**Scheme** **1**a). PMNAs are demonstrated to exhibit a scattering percentage of ≈90% in the second NIR window, which imparts strong OCT signals and contrast. Furthermore, when exposed to the external magnetic field, PMNAs are aggregated via assembly into a chain structure via magnetostatic coupling^[^ ^33^, ^34^, ^35^, ^36^ ^]^ between neighboring PMNAs, which decreases optical scattering cross‐sections at NIR wavelengths and thus reduces OCT signals (Scheme 1b,c). Upon removal of the magnetic field, the assembled PMNAs are disassembled into a dispersed state, thereby restoring their OCT signals (Scheme 1c). The reversible magnetic field‐mediated control of OCT signals from PMNAs allows them to serve as a tool to enhance particle‐associated signal detection with suppressed background signal via image subtraction, which has been demonstrated through in vitro and in vivo experiments (Scheme 1d).

## 2 Results and Discussion

### 2.1 PMNAs are Reversibly Assembled into Chains and Disassembled Under the Modulation of an External Magnetic Field, Regulating Their Optical Responses at NIR Wavelengths

**Figure 1:**
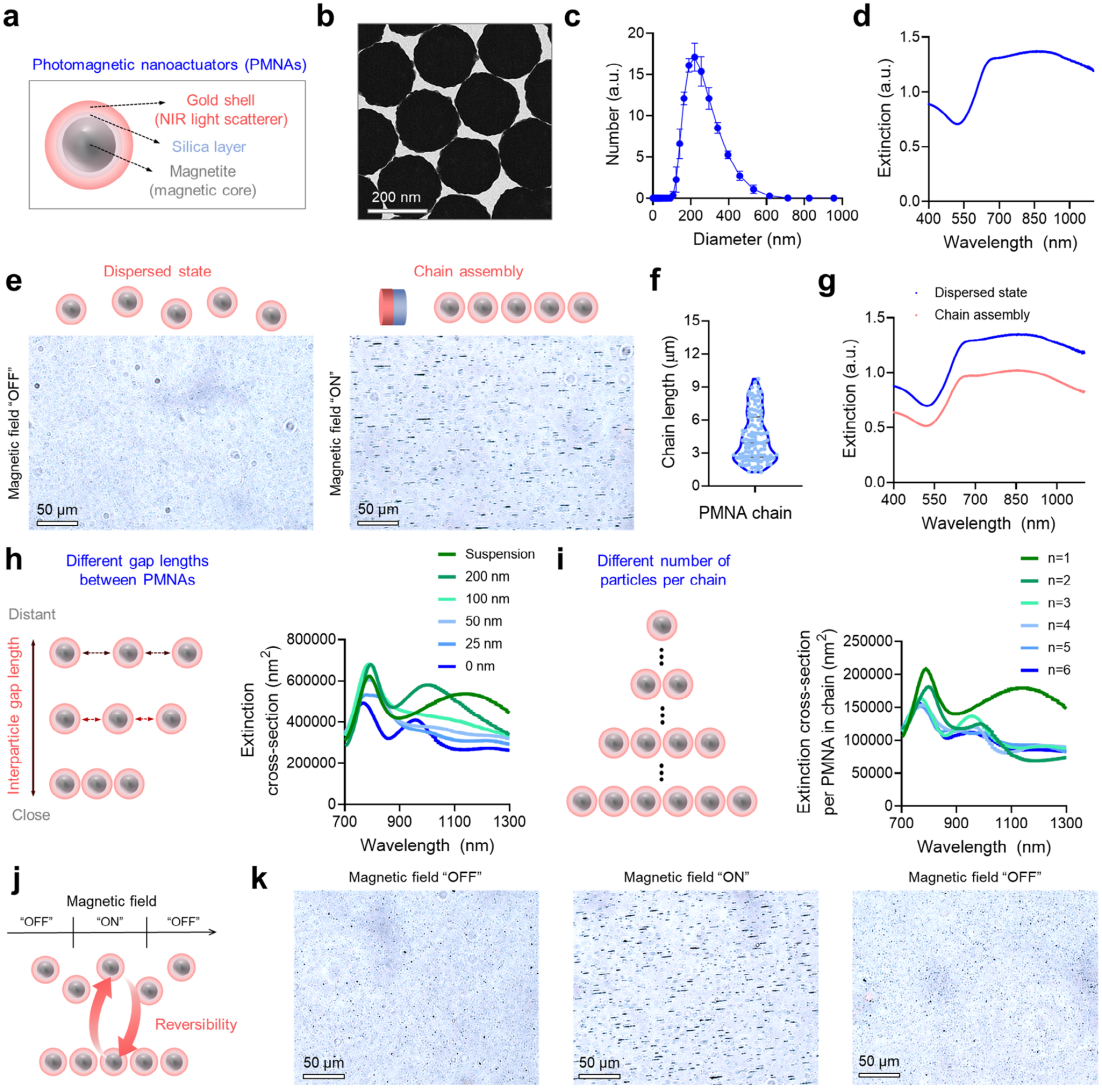
PMNAs are reversibly assembled into chains and disassembled under the modulation of an external magnetic field, regulating their optical responses at NIR wavelengths. a,b) Schematic illustration and the corresponding TEM image of the structure of PMNAs. c) Hydrodynamic diameter distribution of PMNAs in water (*n* = 3). d) UV–vis–NIR spectrum of PMNAs. e) Optical microscope images of PMNAs in water (160 µg mL^−1^) upon the application of an external magnetic field. f) Chain length of PMNAs upon exposure to the external magnetic field (*n* = 115). g) UV–vis–NIR spectra of PMNAs under the control of an external magnetic field. h,i) Calculated extinction cross‐sections of PMNAs at different interparticle gap lengths and different numbers of PMNAs per chain. j,k) Schematic illustration and corresponding optical microscope images of reversible chain assembly and disassembly of PMNAs in water (160 µg mL^−1^) upon the modulation of the external magnetic field. Data are presented as the mean ± standard deviation. The imaging experiments were repeated independently three times, and similar imaging results were obtained.

The intensity of exogenous contrast agents in optical scattering is directly associated with the contrast‐enhanced OCT signals in the NIR window.^[^ ^1^, ^2^, ^3^, ^4^, ^17^, ^18^ ^]^ Thus, we fabricated a magnetic–photonic core–shell nanoconstruct that consists of magnetite (Fe_3_O_4_) core and gold shell, functioning as a magnetic actuator and NIR light scatterer, respectively (**Figure** **1**a; Figure S1a, Supporting Information). Specifically, we synthesized 130 nm‐sized Fe_3_O_4_ nanospheres by a modified polyol method,^[^ ^37^ ^]^ followed by coating with an amine‐functionalized silica (SiO_2_) layer through the Stöber method^[^ ^38^ ^]^ (Figure S1b, Supporting Information). Next, we conjugated 2 nm‐sized gold seeds to the amine‐functionalized Fe_3_O_4_‐SiO_2_ nanospheres via electrostatic interaction between positively charged amine groups and negatively charged gold nanospheres^[^ ^39^ ^]^ (Figure S1b, Supporting Information). Lastly, we carried out conformal shell growth of 2 nm‐sized gold seeds^[^ ^40^ ^]^ on Fe_3_O_4_‐SiO_2_ nanospheres to yield PMNAs based on the mild reduction of gold ions in the presence of formaldehyde. The PMNAs exhibited a diameter of ≈210 nm with structural homogeneity (Figure 1b,c). Due to the gold shell layer,^[^ ^41^, ^42^ ^]^ PMNAs exhibited strong optical responses at NIR wavelengths (Figure 1d). The scattering percentage of the PMNAs was ≈90% in the second NIR window as calculated by finite‐difference time‐domain (FDTD) simulation (Figure S2, Supporting Information).

Next, we investigated whether PMNAs can exhibit magnetic responses and subsequently magnetic field‐induced changes in their optical responses. When exposed to an external magnetic field, PMNAs were assembled into a chain assembly with a length of ≈4.5 ± 2.2 µm due to magnetostatic coupling^[^ ^33^, ^34^, ^35^, ^36^ ^]^ between neighboring PMNAs (Figure 1e,f; Figure S3, Supporting Information). Specifically, upon the external magnetic field excitation, PMNAs exhibit the magnetic interactions between neighboring particles (i.e., magnetostatic coupling) due to the presence of Fe_3_O_4_ core, which overcomes thermal fluctuations and electrostatic repulsion between nearby PMNAs, thereby yielding a chain assembly along the magnetic field direction^[^ ^33^, ^36^, ^43^, ^44^ ^]^ (Figure S4, Supporting Information). Furthermore, we observed that higher magnetic field strength and longer exposure durations led to the formation of longer chains, compared to weaker fields and shorter exposure times (Figures S5 and S6, Supporting Information). Collectively, these results demonstrate that both magnetic field intensity and treatment duration are critical parameters in modulating PMNA chain assembly, as they regulate the balance between attractive dipole–dipole interactions and interparticle/interchain repulsion.

The magnetic chain assembly resulted in a decrease in the optical extinction of PMNAs compared to their dispersed suspension state (Figure 1g). As shown by transmission electron microscopy, PMNAs formed 1D chain structures with reduced interparticle spacing under external magnetic field excitation (Figure S3, Supporting Information). To investigate this phenomenon, we numerically estimated the extinction cross‐sections of linearly arranged PMNAs, varying the interparticle gap length while keeping the number of particles constant at three. Our analysis revealed that as the interparticle distance between adjacent PMNAs was reduced, the intensity of extinction cross‐sections also decreased (Figure 1h), which aligns with our findings from the UV–vis–NIR spectroscopy (Figure 1g). Moreover, we investigated whether the number of PMNAs per chain could affect the change of optical responses at NIR wavelengths. Specifically, we characterized extinction cross‐sections per PMNA in chains with varying numbers of PMNAs per chain (Figure 1i). We found that, when PMNAs are assembled into a chain construct, the extinction cross‐section per PMNA in a chain decreased compared to that in the dispersed state of PMNAs (Figure 1i).

Additionally, the assembly and disassembly of PMNAs were reversibly controlled by modulating the magnetic field applied to their colloidal suspension (Figure 1j,k). The disassembly of PMNAs could be explained by the repulsive electrostatic interaction of the particles^[^ ^36^, ^45^ ^]^ and their Brownian motion.^[^ ^46^ ^]^ The magnetic chain assembly behavior of PMNAs was further validated in viscous media with viscosities ranging from ≈0.9 to 400 cP, achieved by adjusting the glycerol volume fractions in water (Figure S7, Supporting Information). Notably, the viscosities of various physiological environments—such as intracellular regions,^[^ ^47^ ^]^ interstitial fluid,^[^ ^48^ ^]^ blood,^[^ ^49^ ^]^ plasma,^[^ ^49^ ^]^ vitreous humor,^[^ ^50^ ^]^ and bone marrow^[^ ^51^ ^]^—typically fall within this range, indicating that PMNAs maintain effective responsiveness to magnetic modulation under physiologically relevant conditions. Moreover, PMNAs demonstrated reversible chain assembly and disassembly in a viscous medium with a viscosity comparable to that of blood^[^ ^49^ ^]^ (Figure S8, Supporting Information). Taken together, these results suggest that PMNAs could be used to remotely modulate OCT signals and contrast by dynamically controlling an external magnetic field.

### 2.2 PMNAs Function as a Dynamic OCT Contrast Agent with Magnetic Field‐Mediated Signal Modulation

**Figure 2:**
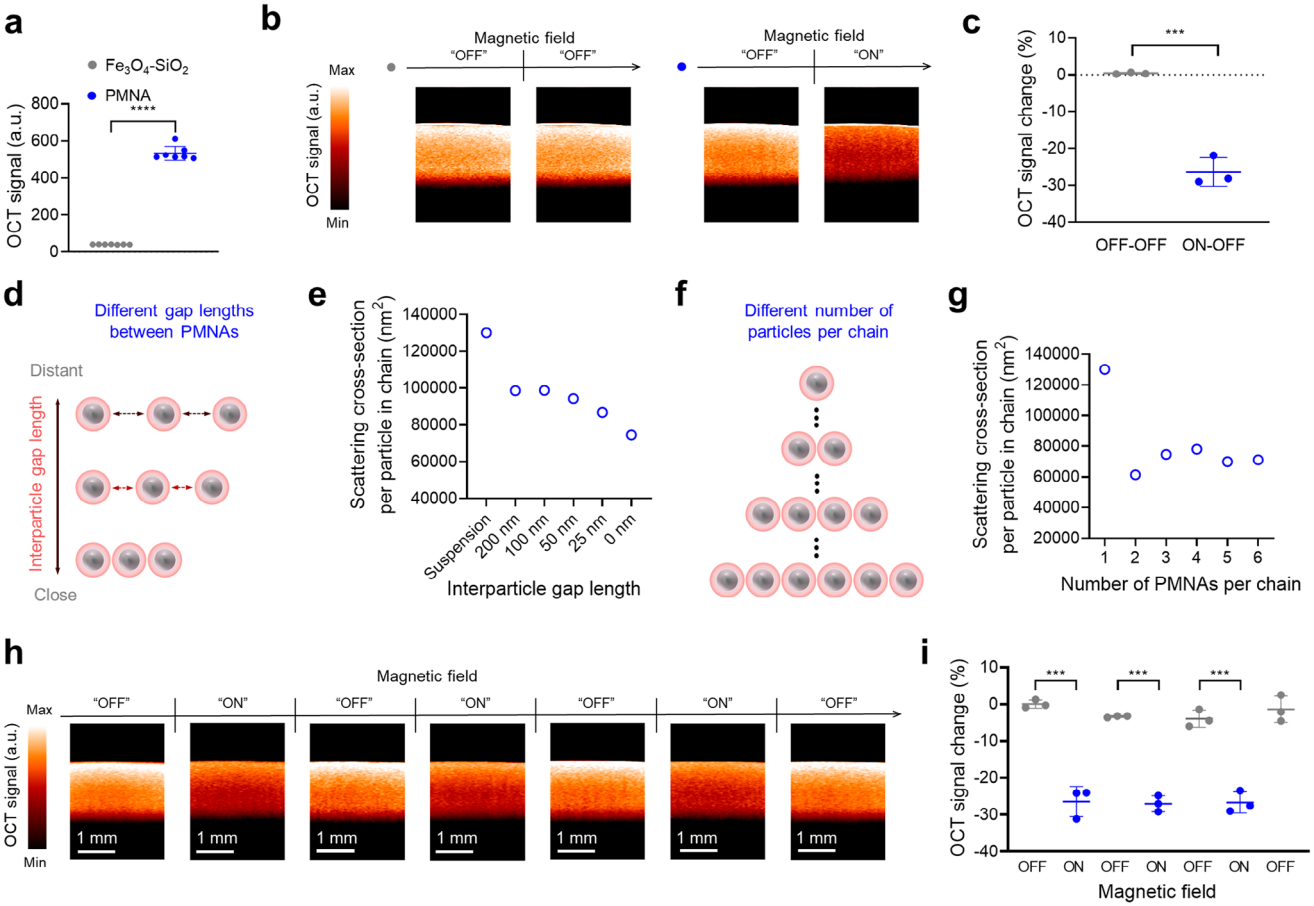
PMNAs function as a dynamic OCT contrast agent with magnetic field‐mediated signal modulation. a) OCT signal generation from Fe_3_O_4_‐SiO_2_ core–shell nanospheres and from PMNAs (*n* = 7). b,c) Changes in OCT signals from PMNAs (320 µg mL^−1^) by modulating the external magnetic field (*n* = 3). d,e) Calculated scattering cross‐section of the PMNA chain with different interparticle gap lengths at a wavelength of 1300 nm. f, g) Calculated scattering cross‐section per particle of the PMNA chain with different PMNA numbers at a wavelength of 1300 nm. h, i) Reversible modulation of OCT signals from PMNAs (320 µg mL^−1^) by modulating the external magnetic field (*n* = 3). Data are presented as the mean ± standard deviation. The statistical analysis for Figure 2a,c was conducted using a two‐tailed Student's *t*‐test. The statistical analysis for Figure 2i was conducted using a one‐way ANOVA with Tukey post‐hoc test. The statistically significant difference is represented as the asterisk (***: *p* < 0.001, ****: *p* < 0.0001). The imaging experiments were repeated independently three times, and similar imaging results were obtained.

We sought to demonstrate that the reversible assembly and disassembly of PMNAs with changes of optical responses at NIR wavelengths under the control of an external magnetic field could be utilized to remotely and repeatably modulate OCT signals and contrast. We first characterized OCT signals from the PMNA solution at a wavelength of 1300 nm via a spectral‐domain OCT system (Figure S9, Supporting Information), while comparing those with Fe_3_O_4_‐SiO_2_ nanospheres. We observed significantly higher OCT signals from PMNAs compared to Fe_3_O_4_‐SiO_2_ nanospheres, owing to the contribution of the gold shell layer to the optical scattering (**Figure** **2**a; Figure S10, Supporting Information). The depth‐dependent OCT signal profile of PMNAs revealed a decrease in signal amplitude with increasing depth, due to light attenuation caused by the presence of PMNA (Figure S11, Supporting Information). Furthermore, PMNAs exhibited concentration‐dependent OCT signal generation, with detectable signals observed even at a concentration as low as 0.625 µg mL^−1^ (Figure S12, Supporting Information). Additionally, PMNAs produced reliable OCT signals across different imaging sessions, ensuring their photostability (Figure S13, Supporting Information).

Next, we investigated whether the magnetic chain assembly could change OCT signals from the PMNA solution. In the absence of an external magnetic field (“OFF” state), OCT signals and contrast from the PMNA solution were not changed (Figure 2b,c). However, when the external magnetic field was applied to the PMNA solution (“ON” state), the OCT signals were reduced by ≈30% with decreased image contrast (Figure 2b,c). Furthermore, we observed that particle aggregation led to ≈35% decrease in OCT signal, indicating that nanoparticle aggregation can reduce optical scattering and, consequently, OCT responses (Figure S14, Supporting Information). Nevertheless, magnetic field‐mediated chain‐like aggregation offers a more controllable strategy for modulating OCT signals from PMNAs, as the aggregates can be readily disassembled upon removal of the magnetic field. Additionally, we found that either increased magnetic field strength or longer exposure durations resulted in larger changes in OCT signals (Figures S15 and S16, Supporting Information). This suggests that both magnetic field parameters play a crucial role in modulating OCT signals by controlling the chain length of PMNAs (Figures S5 and S6, Supporting Information).

We further assessed whether medium viscosity affects the performance of PMNAs in magnetic field‐mediated OCT signal modulation. The results showed that PMNAs exhibited significant OCT signal differences between the “OFF” and “ON” states across a viscosity range of ≈0.9 to 400 cP, which falls within the typical viscosity range of physiological environments^[^ ^47^, ^48^, ^49^, ^50^, ^51^ ^]^ (Figure S17, Supporting Information). Moreover, PMNAs immobilized in a solidified medium showed no change in OCT signal under the external magnetic field excitation (Figure S18, Supporting Information). This result indicates that the free motion of PMNAs is a critical factor to achieve magnetically controlled OCT signal modulation.

As OCT signal generation from the PMNA solution is determined by the particle scattering intensity,^[^ ^1^, ^2^, ^3^, ^4^, ^17^, ^18^ ^]^ we explored the effect of the magnetic field‐mediated chain assembly of PMNAs on optical scattering responses. We first investigated the influence of interparticle distances between PMNAs in the chain assembly on the optical scattering intensity of PMNAs at 1300 nm (Figure 2d). We numerically calculated optical scattering cross‐sections per PMNA of linearly placed PMNAs (number of particles = 3) with different interparticle gap distances at 1300 nm via a FDTD simulation (Figure 2e). We observed that, as the interparticle distance between adjacent PMNAs decreased, the scattering cross‐section also decreased. Next, we further investigated the influence of the number of PMNAs per chain on the optical scattering intensity (Figure 2f). In detail, through the FDTD computational analysis, we numerically evaluated scattering cross‐sections per PMNA in a chain with different numbers of particles while maintaining the interparticle distance identical. The computational result showed that, when PMNAs are assembled into a chain construct, the scattering cross‐sections per PMNA are reduced (Figure 2g). Overall, the results indicated that magnetic field‐mediated chain assembly of PMNAs leads to the reduction of the optical scattering responses, thereby decreasing their OCT signals and contrast.

Given the ability of PMNAs to reversibly assemble and disassemble in response to an external magnetic field, we investigated whether OCT signals and contrast from the PMNA solution could be repeatably modulated by magnetically controlling their assembly and disassembly behavior. To verify this, we characterized OCT signals from the PMNA suspension upon magnetic field perturbation across three repeated “OFF”‐“ON” cycles (Figure 2h,i). In the presence of the magnetic field, PMNAs exhibited a reduction of OCT signals by ≈30% due to the decreased optical scattering upon chain assembly. Upon removal of the magnetic field, the OCT signals recovered to their original state, demonstrating that PMNAs can be utilized toward remote and reversible modulation of OCT signal generation under controlled magnetic field excitation (Figure 2i). The reversibility of OCT signal changes of PMNAs was further validated in a viscous medium with a viscosity comparable to that of blood^[^ ^49^ ^]^ (Figure S19, Supporting Information). These findings highlight the potential of PMNAs as dynamic OCT contrast agents with magnetic field‐mediated signal modulation.

### 2.3 Magnetic Modulation of OCT Signals from PMNAs is Utilized for Identifying Particle‐Associated Signals In Vitro

**Figure 3:**
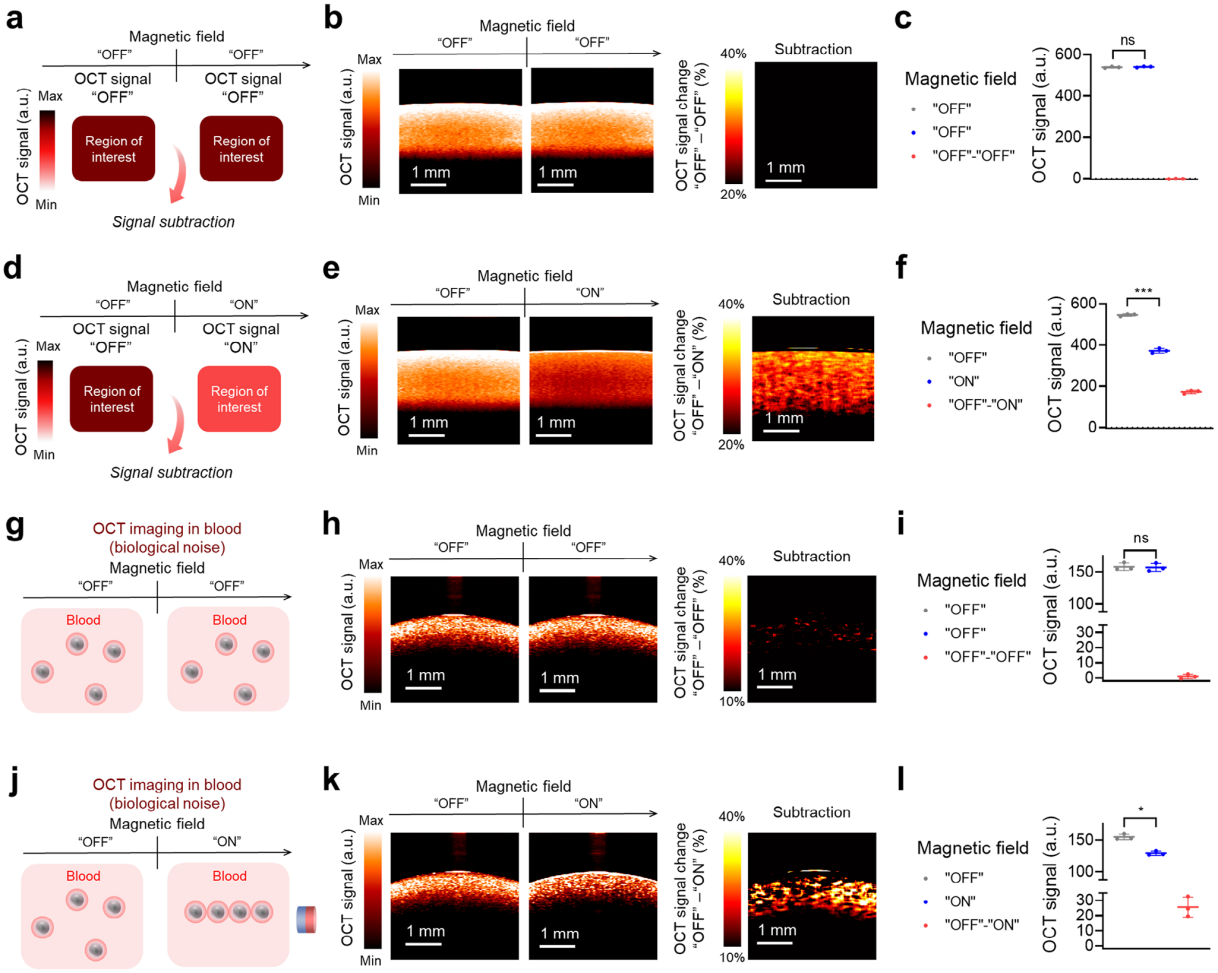
Magnetic modulation of OCT signals from PMNAs is utilized for identifying particle‐associated signals in vitro. a–c) OCT signal generation from PMNAs (320 µg mL^−1^) in the water solution without the modulation of the external magnetic field (*n* = 3). d–f) OCT signal generation from PMNAs (320 µg mL^−1^) in the water solution with the modulation of the external magnetic field (*n* = 3). g–i) OCT signal generation from PMNAs (213 µg mL^−1^) in blood without the modulation of the external magnetic field (*n* = 3). j–l) OCT signal generation from PMNAs (213 µg mL^−1^) in blood with the modulation of the external magnetic field (*n* = 3). Data are presented as the mean ± standard deviation. The statistical analysis for Figure 3c,f,i,l was conducted using a two‐tailed Student's *t*‐test. The statistically significant difference is represented as the asterisk (ns: non‐significant, *: *p* < 0.05, ***: *p* < 0.001). The imaging experiments were repeated independently three times, and similar imaging results were obtained.

As PMNAs modulate their OCT signals and contrast upon the controlled excitation of an external magnetic field, we sought to demonstrate whether the OCT signal changes between “ON” and “OFF” states could be employed for identifying particle‐associated signals with specificity (**Figure** **3**). Explicitly, we subtracted OCT signals from PMNAs in the “OFF” state from those in the “ON” state and quantified the signal difference between the two states. Without perturbation of the external magnetic field (i.e., “OFF” to “OFF” states), OCT signals from the PMNA solution remained consistent, showing no significant difference between the two individual “OFF” modes (Figure 3a–c). By contrast, when the external magnetic field was applied to the PMNA solution from “OFF” to “ON” states, we observed ≈30% of OCT signal change between “OFF” and “ON” modes (Figure 3d–f). Moreover, the signal‐to‐noise ratio in the differential “OFF”‐“ON” mode decreased with depth at a much lower rate than the original OCT signal from PMNAs (Figure S20, Supporting Information). These results suggest that the magnetic field‐mediated modulation of OCT responses from PMNAs enhances the detection of particle‐associated signals with improved imaging depth.

Next, we investigated whether the magnetic field‐modulated OCT signal changes of PMNAs can be implemented to specifically identify the imaging signals from PMNAs in a fluid‐like tissue specimen, blood (Figure 3g–l). We first observed that blood alone does not exhibit any OCT signal changes in the presence of the external magnetic field (Figure S21, Supporting Information). This result indicated that endogenous hemoglobin does not exhibit any OCT signal changes upon the external magnetic field application. To improve the colloidal stability of PMNAs in blood, we functionalized polyethylene glycol (PEG) to the surface of PMNAs^[^ ^52^ ^]^ via a gold‐thiol bond,^[^ ^53^, ^54^ ^]^ as validated by Zeta potential analysis (Figure S22, Supporting Information). We validated that the PEGylation process does not compromise the performance of OCT responses and signal changes under the modulation of the external magnetic field, while ensuring the signal stability at different pH conditions (Figures S23 and S24, Supporting Information). When PMNAs were dispersed in blood, we observed a significant OCT signal increase due to the contribution of PMNAs to enhanced optical scattering (Figure S25, Supporting Information). In the absence of an external magnetic field (“OFF” to “OFF” states), there was no detectable difference in OCT signals from PMNAs in blood between successive “OFF” states (Figure 3g–i). However, upon the application of the external magnetic field, we observed a significant change in OCT signals by modulating the state of PMNAs from “OFF” to “ON”, indicating the presence of PMNAs in blood (Figure 3j–l). Furthermore, we demonstrated that OCT signal generation and modulation by PEGylated PMNAs in response to the external magnetic field are unaffected by the presence of proteases or mouse splenocytes, suggesting the potential of PMNAs for applications in biological systems (Figure S26, Supporting Information). Collectively, our results demonstrate the potential of magnetic field‐mediated OCT signal modulation of PMNAs for the identification of particle‐associated signals.

### 2.4 Magnetic Modulation of OCT Responses of PMNAs is Leveraged to Enhance Particle‐Associated Signal Detection with Suppressed Background Signal In Vivo

**Figure 4:**
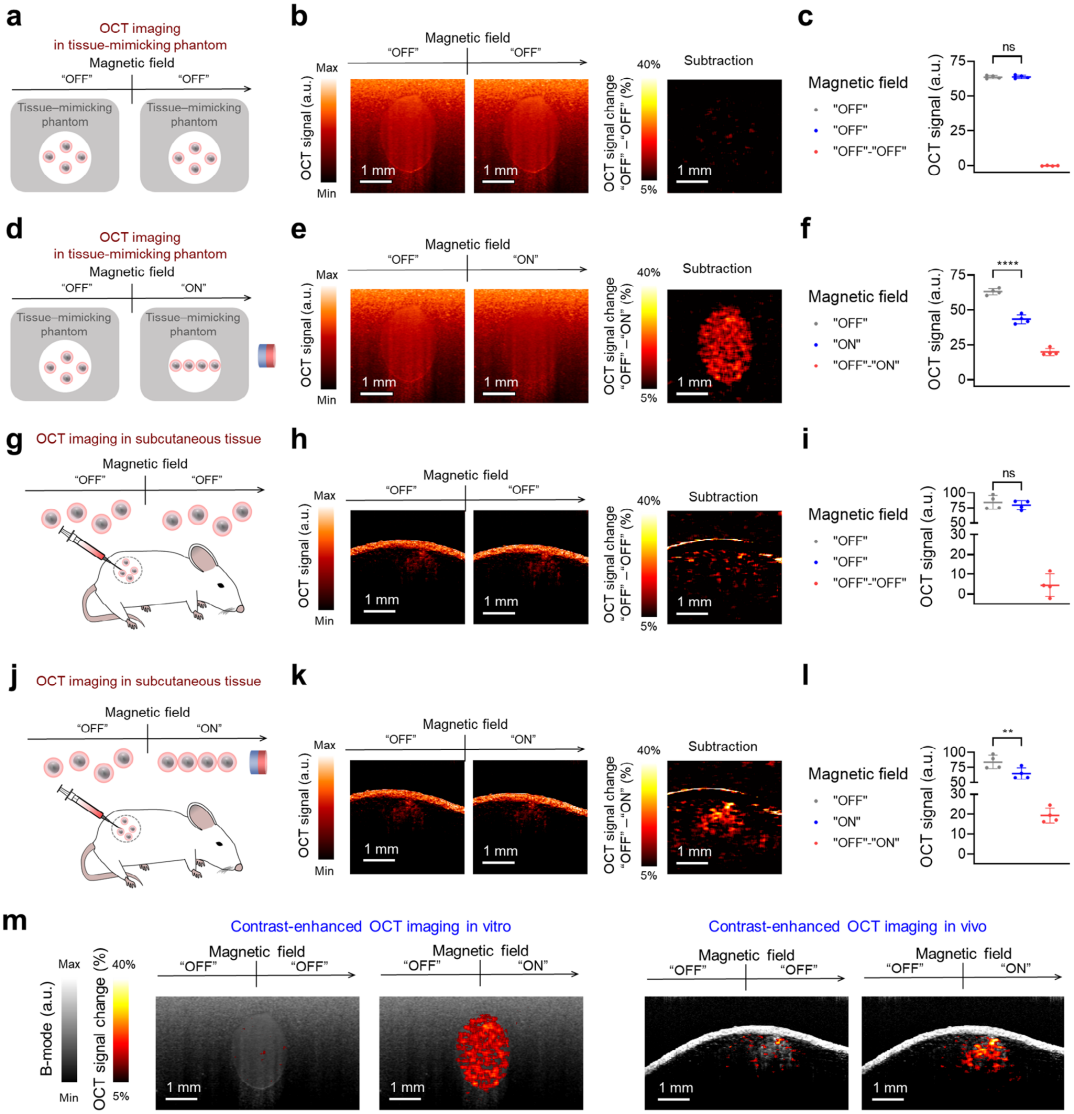
Magnetic modulation of OCT responses of PMNAs is leveraged to enhance particle‐associated signal detection with suppressed background signal in vivo. a–c) OCT signal generation from the PMNA inclusion (580 µg mL^−1^) in the tissue‐mimicking phantom without the modulation of the external magnetic field (*n* = 4). d–f) OCT signal generation from the PMNA inclusion (580 µg mL^−1^) in the tissue‐mimicking phantom with the modulation of the external magnetic field (*n* = 4). g–i) OCT signal generation from PMNAs (20 µg) in the mouse tissue after subcutaneous particle injection without the modulation of the external magnetic field (*n* = 4). j–l) OCT signal generation from PMNAs (20 µg) in the mouse tissue after subcutaneous particle injection with the modulation of the external magnetic field (*n* = 4). m) Overlayed OCT B‐mode and subtraction images under the modulation of an external magnetic field, both in vitro and in vivo. Data are presented as the mean ± standard deviation. The statistical analysis for Figure 4c,f,i,l was conducted using a two‐tailed Student's *t*‐test. The statistically significant difference is represented as the asterisk (ns: non‐significant, *: *p* < 0.05, **: *p* <0.01, ***: *p* <0.001). The imaging experiments were repeated independently four times, and similar imaging results were obtained.

As magnetic field‐mediated assembly of PMNAs can be used to specifically identify the presence of PMNAs in OCT, we went on to demonstrate the potential of PMNAs for enhanced particle‐associated signal detection with suppressed background signal via pixel‐by‐pixel subtraction of pre‐ and post‐magnetic field treatment images. First, we prepared a tissue‐mimicking gelatin phantom containing titanium oxide as a light scatterer^[^ ^55^ ^]^ for in vitro characterization of the background subtraction. We first observed that titanium oxide itself in the gelatin phantom did not change OCT signals and image contrast upon the control of an external magnetic field (Figure S27, Supporting Information). Next, we embedded a tube‐shaped inclusion of PMNAs inside the tissue‐mimicking phantom and carried out OCT while perturbating an external magnetic field to control the chain assembly of PMNAs. Without applying the external magnetic field, we observed no OCT signal changes across consecutive imaging frames, i.e., different “OFF” modes (**Figure** **4**a–c). Conversely, when we applied the magnetic field to the PMNA inclusion (“OFF” to “ON”), we observed OCT signal changes with a 30% reduction in OCT signal, thereby enhancing particle‐associated signal detection with suppressed background signal through subtraction of images before and after external magnetic field applications (Figure 4d–f).

Lastly, we demonstrated the capability of magnetically controlled OCT signals from PMNAs to achieve enhanced particle‐associated signal detection in vivo by performing subcutaneous injections into healthy mice. We first found that our PMNAs did not exhibit any cytotoxicity against mouse splenocytes, which include various host immune cells, up to a concentration of 174 µg mL^−1^ (Figure S28, Supporting Information). While tissue alone and tissue injected with saline did not generate any noticeable changes in OCT signals and contrast upon the external magnetic field treatment (Figures S29 and S30, Supporting Information), mice that received PMNAs displayed significant OCT signal changes under the external magnetic field application, enabling the detection of OCT signals from PMNAs with suppressed background signal via post processing with image subtraction (Figure 4g–l). We further estimated the contrast‐to‐noise ratio (CNR) in the region of interest where particles were injected, specifically in the “OFF”‐“ON” mode, and compared it with the CNR values from B‐mode imaging (i.e., prior to image subtraction) and the “OFF”‐“OFF” mode. The results showed that the CNR in the “OFF”‐“ON” mode was significantly higher than in the other modes (Figure S31, Supporting Information). Additionally, we assessed the enhancement of particle‐associated signal detection by quantifying precision values for both B‐mode and the differential “OFF”‐“ON” mode images. The differential mode yielded significantly higher precision values compared to B‐mode imaging (Figure S32, Supporting Information). The capability of the enhanced particle‐associated signal detection with magnetically controlled OCT responses of PMNAs is further illustrated by showing overlayed OCT B‐mode and differential images under the modulation of an external magnetic field both in vitro and in vivo (Figure 4m). Finally, we confirmed the reversibility of OCT signal changes from PMNAs in vivo across multiple “OFF”‐“ON” cycles (Figure S33, Supporting Information). While physiological motion artifacts, such as respiration, could introduce residual background signals despite pixel‐by‐pixel subtraction of images acquired before and after magnetic field modulation, our results demonstrate the effectiveness of PMNAs for contrast‐enhanced OCT through magnetic field‐mediated signal control. Future studies could include the implementation of real‐time or retrospective motion compensation techniques, either during data acquisition or in post‐processing, to improve the suppression of the background signal. Furthermore, we plan to investigate optimized structural parameters of PMNAs, such as magnetite core diameter, gold shell thickness, and overall size and shape of the actuator in combination with more comprehensive numerical analyses to tailor magnetic, optical, and physicochemical properties for a variety of biomedical applications requiring enhanced OCT contrast.

## 3 Conclusion

We fabricated PMNAs with a scattering efficiency of 90%, comprising of magnetite core and a gold shell, which function as a magnetic motor and a NIR light scatterer, respectively. This structure enables PMNAs to modulate their optical scattering in the second NIR window and OCT signals under the control of an external magnetic field. Specifically, when exposed to the external magnetic field, PMNAs were assembled into chain‐like structures, leading to a decrease in optical scattering intensity and a corresponding reduction of OCT signals and contrast. Upon removal of the magnetic field, the assembled PMNAs return to a monodispersed state, thus restoring the scattering responses and corresponding OCT signals. This behavior of PMNAs under modulated magnetic field excitations demonstrates the ability to remotely and reversibly control OCT signal generation. We utilized these two distinct assembly and disassembly states of PMNAs to enhance particle‐associated signal detection with suppressed background signal by simply performing pixel‐by‐pixel subtraction of images before and after the external magnetic field treatment, both in vitro and in vivo. The demonstrated nanoactuator platform offers a promising strategy for contrast‐enhanced OCT. Furthermore, since the performance of our nanoactuator platform is independent of tissue mechanical properties, it has the potential to broaden the applications of OCT in noninvasive cell monitoring and molecular diagnostic imaging in preclinical animal models. Potential applications include stem cell monitoring and tracking through PMNA labeling for ophthalmological studies, as well as tumor imaging using targeting ligand‐functionalized PMNAs.

## 4 Experimental Section

### Synthesis of Magnetite Nanospheres

Magnetite (Fe_3_O_4_) nanospheres were synthesized by a previously reported modified polyol method.^[^ ^37^ ^]^ First, 0.5406 g of iron chloride hexahydrate (Sigma–Aldrich), 0.4922 g of sodium acetate (Sigma–Aldrich), and 2.703 g of deionized water were dissolved in 50 mL of ethylene glycol (Sigma–Aldrich), followed by magnetic stirring for 15 min at room temperature. Next, the mixture was heated up to 200 °C for 4 h. The fabricated Fe_3_O_4_ nanoparticles were washed with ethanol three times via centrifugation (9000 rpm for 10 min), followed by dispersing in ethanol (10 mg mL^−1^).

### Silica Layer Coating on Fe_3_O_4_ Nanospheres and Amine Functionalization

Fe_3_O_4_ nanospheres were covered with a silica layer via a modified Stöber method.^[^ ^38^ ^]^ Specifically, 20 mg of Fe_3_O_4_ nanospheres was dissolved in 40 mL of ethanol, 2 mL of ammonium hydroxide solution (28% NH_3_ in H_2_O, Sigma–Aldrich), and 6 mL of deionized water. The mixture was mechanically stirred for 5 min. To this solution, 0.04 mL of tetraethyl orthosilicate (Sigma–Aldrich) was added, followed by stirring overnight. The silica‐coated Fe_3_O_4_ nanospheres were washed with ethanol three times via centrifugation (9000 rpm for 10 min) and then dispersed in 20 mL of ethanol. For amine functionalization, 10 mL of silica‐coated Fe_3_O_4_ (Fe_3_O_4_‐SiO_2_) nanospheres in ethanol was mixed with 1.5 mL of deionized water, 0.5 mL of ammonium hydroxide solution, and 0.1 mL of (3‐aminopropry)triethoxysilane (Sigma–Aldrich). The mixture was then stirred overnight to yield amine groups on the surface of Fe_3_O_4_‐SiO_2_ nanospheres. The amine‐functionalized particles were then washed with ethanol and water three times, respectively, via centrifugation (9000 rpm for 10 min), followed by dispersing them in 1 mL of deionized water.

### Synthesis of 2 nm‐Sized Gold Nanospheres

Gold nanospheres (2 nm) were synthesized as reported previously.^[^ ^39^ ^]^ Specifically, 0.664 mL of 1 m sodium hydroxide (Sigma–Aldrich) and 0.016 mL of tetrakis(hydroxymethyl)phosphonium chloride (Sigma–Aldrich) were added to 60 mL of deionized water. To this solution, 3.84 mL of 25 mm gold chloride trihydrate (Sigma–Aldrich) was quickly added, followed by magnetic stirring for 5 min. The product was stored at 4 °C in the dark for further use.

### Conjugation of 2 nm‐Sized Gold Nanospheres to the Surface of Amine‐Functionalized Fe_3_O_4_‐SiO_2_ Nanospheres

As gold nanoparticles can bind to the amine groups on Fe_3_O_4_‐SiO_2_ nanospheres via electrostatic interaction,^[^ ^39^ ^]^ 1 mL of amine‐functionalized silica‐coated Fe_3_O_4_ nanospheres was mixed with 30 mL of 2 nm‐sized gold nanospheres, followed by sonication for 10 min at room temperature. The gold nanosphere‐conjugated magnetic nanospheres were washed with deionized water twice and then dispersed in 12 mL of deionized water.

### Preparation of Gold Shell Growth Solution

The gold shell growth solution was prepared by mixing 6 mL of 25 mm gold chloride trihydrate and 100 mg of potassium carbonate (Sigma–Aldrich) with 794 mL of deionized water.^[^ ^39^ ^]^ The mixture was magnetically stirred overnight in the dark.

### Gold Shell Growth to Yield PMNAs

To fabricate PMNAs, 2.5 mL of gold nanosphere‐conjugated Fe_3_O_4_‐SiO_2_ nanospheres was dispersed in 800 mL of the as‐prepared gold growth solution, followed by magnetic stirring for 5 min. Next, 1 mL of formaldehyde solution (Sigma–Aldrich) was quickly added, followed by magnetic stirring for 1 h. To this solution, 5 mL of 5 wt.% polyvinylpyrrolidone aqueous solution (Sigma–Aldrich) was added to stabilize the fabricated PMNAs. The stabilized PMNAs were washed with deionized water twice via centrifugation (4000 rpm for 20 min). To functionalize polyethylene glycol (PEG) ligands onto the surface of PMNAs, the fabricated PMNAs in 1 mL of deionized water were mixed with 0.2 mL of 1 mm carboxylmethyl‐PEG‐thiol (Laysan Bio, Inc., molecular weight 5000), followed by shaking overnight in the dark. The PEGylated PMNAs were washed with deionized water via centrifugation twice (6000 rpm for 10 min) and then dispersed in 1 mL of phosphate‐buffered saline (PBS).

### Characterization for PMNAs

Transmission electron microscopy (HT 7700, Hitachi) was utilized to demonstrate the serial steps in the synthesis of PMNAs. The optical characteristics of PMNAs were characterized by a UV–vis–NIR spectrophotometer (Evolution 220, Theormoscientific). The hydrodynamic diameter distribution and surface charges of photomagnetic nanoactuators were characterized using a dynamic light scattering instrument (Zetasizer Nano ZS, Malvern Instruments Ltd.).

### Numerical Simulation

The optical cross‐sections, such as extinction, absorption, and scattering, for PMNAs were calculated by a finite‐difference time‐domain (FDTD) simulation (Lumerical Inc.). For the FDTD analysis, the surrounding temperature was set as 300 K. The surrounding medium was set as water. The optical characteristics of gold, silica, and magnetite, such as dielectric constant and refractive index, were taken from the values in Johnson and Christy,^[^ ^56^ ^]^ Palik,^[^ ^57^ ^]^ and Querry,^[^ ^58^ ^]^ respectively. To compute the optical cross‐sections, we utilized a total‐field/scattered (TF/SF) source with a wavelength range from 700 to 1300 nm for PMNAs with a diameter of 210 nm as characterized by TEM. To investigate the effect of the chain assembly of PMNAs on optical scattering cross‐sections, the number and interparticle gap length of PMNAs per chain were controlled. Furthermore, a maximum mesh step of 1.8 nm was set for calculating the optical characteristics of PMNAs. To obtain extinction, absorption, and scattering cross‐sections for PMNAs, the average values were calculated from light excitation at different light polarization directions, including p‐ and s‐polarization.

### Characterization for OCT Signal Generation from PMNAs

OCT data were collected using a commercial spectral‐domain OCT system (TEL221PSC1/M, ThorLabs Inc.). The system used a broadband light source with a center wavelength of 1300 nm and a bandwidth of 170 nm, providing an axial resolution of ≈5.5 µm in air. Imaging was conducted with an objective lens (LSM03, ThorLabs Inc.) that provided a lateral resolution of 13 µm. The system features a high‐speed spectrometer, enabling a 76 kHz A‐scan rate with 96 dB sensitivity. For each acquisition, 20 B‐mode images were acquired within 18 ms, with two consecutive frames averaged to enhance the signal‐to‐noise ratio (SNR). To enable magnetic control of OCT signals from PMNAs, a neodymium magnet (NR0082‐45NM, CMS Magnetics) was used to apply a magnetic field strength of ≈100 mT to the PMNAs.

To measure OCT signals from particle suspensions, we added the aqueous solution containing PMNAs or Fe_3_O_4_‐SiO_2_ into a 96‐well plate and then acquired OCT signals from the particle solution. To assess OCT signals from PMNAs in the viscous medium, PMNAs were dispersed in glycerol (Sigma‐Aldrich)‐water mixtures with varying glycerol volume fractions of 0%, 12.5%, 25%, 50%, 75%, and 95%. To acquire OCT signals from PMNAs in the presence of different proteases, PMNAs were dispersed in 17 nm recombinant murine granzyme B (GzmB, ReproTech), recombinant human cleaved caspase‐3 (Cas‐3, abcam), matrix metalloproteinase‐9 (MMP‐9, BioVision), matrix recombinant human metalloproteinase‐7 (MMP‐7, BioLegend), or thrombin (Sigma–Aldrich) in PBS solution.

All animal experiments were performed under the Institutional Animal Care and Use Committee (IACUC, protocol number: A100281) guidelines of the Georgia Institute of Technology. Blood samples were directly isolated from blood vessels in healthy mice (The Jackson Laboratory, female, 4‐ to 6‐month‐old, C57BL/6J), followed by treating with anticoagulant to inhibit solidification. Next, 20 µL of blood was mixed with 40 µL of the PMNA solution or saline solution to acquire OCT signals and contrast.

Splenocytes were isolated from the spleen in healthy mice (The Jackson Laboratory, 4‐ to 6‐month‐old, female, C57BL/6J). Red blood cells were depleted by treatment with red blood cell lysis buffer (BioLegend). The splenocytes were suspended at a cell density of 2 000 000 cells per mL in media consisting of RPMI‐1640 (Cytvia) with glutamine supplemented with 10% fetal bovine serum (Sigma–Aldrich), 1% penicillin/streptomycin (Sigma–Aldrich), 1% non‐essential amino acid (Thermo Fisher Scientific), 1% sodium pyruvate (Thermo Fisher Scientific), and 0.05 mm 2‐mercaptoethanol (Sigma–Aldrich). Next, 150 µL of splenocytes in the cell culture media or 150 µL of the media were mixed with 150 µL of PMNAs in the media to acquire OCT signals and contrast.

To characterize OCT signals from PMNAs in a tissue‐mimicking gelatin phantom, we fabricated the phantom by adding 400 mg of titanium oxide (Sigma–Aldrich) and 20 g of gelatin powder (MP Biomedicals) into 200 mL of deionized water. The mixture was solidified at 4 °C. To make the inclusion of PMNAs, a tube‐shaped rod was placed into the phantom during the solidification process. Next, PMNAs were gently added to the tube‐shaped region confined in the phantom for characterizing OCT signals and contrast. To assess OCT signals from PMNAs in a solidified gelatin matrix, 50 µL of PMNAs in water was mixed with 50 µL of gelatin aqueous solution (0.1 g mL^−1^). The mixture was solidified at 4 °C for at least 1 h to ensure the solidification process.

### In Vitro Cytotoxicity of PMNAs

Splenocytes (150 000 cells in 100 µL of cell culture media) were dispersed in a 96‐well plate, followed by adding 100 µL of PMNAs at varying concentrations from 0 to 348 µg mL^−1^ and then incubating them for 16 h. Next, the splenocytes were stained with a live/dead agent (ab115347, abcam), and their viability was quantified via flow cytometry (Cytek Aurora).

### In Vivo OCT Characterization for PMNAs

All in vivo OCT experiments were performed under IACUC (protocol number: A100281) guidelines of the Georgia Institute of Technology. The PMNA solution or saline solution was subcutaneously injected into mice (The Jackson Laboratory, 2‐ to 13‐month‐old, female, NU/J). The mice were anesthetized and placed on a heating pad under the OCT system lens. For each acquisition, 20 B‐mode images were acquired within 18 ms, with two consecutive frames averaged to enhance the signal‐to‐noise ratio (SNR). These two frames were selected such that the impact of respiratory motion was neglected.

### Calculation of the Contrast‐to‐Noise Ratio (CNR) and Precision Values

The CNR values were calculated in B‐mode, “OFF”‐“OFF”, and “OFF”‐“ON” mode images to quantify the contrast enhancement via pixel‐by‐pixel subtraction of images before and after magnetic field applications. Using the OCT B‐mode image, a region of interest (ROI) was defined around the site of particle injection, and the mean OCT signal intensity within this region was calculated (I_ROI_). Another ROI was selected in a background region to determine the mean background signal intensity (I_bg_) and the standard deviation of the noise (σ_bg_). CNR was then calculated as CNR = |I_ROI_ −I_bg_|/σ_bg_.

Precision (p)^[^ ^59^ ^]^ was estimated in the OCT images to quantify the particle‐associated signal detection of our method. We defined p = TP⁄(TP+FP), where TP (true positives) denotes the number of pixels correctly classified as particle‐associated signals and FP (false positives) represents the number of pixels in the background incorrectly classified as particle signals. From the OCT B‐mode, an ROI was defined around the area of particle injection (ROI_s_) and a separate ROI in the background region (ROI_bg_). The maximum background noise level was estimated within ROI_bg_ in the subtraction image and defined as the TP threshold. Finally, TP was calculated as the number of pixels inside ROI_s_ in the subtracted image that are above the TP threshold, and TP+FP as the total number of pixels in the entire imaging exceeding the same threshold.

### Software

All imaging experiments were performed independently at least three times. All acquired OCT imaging data were processed and analyzed using MATLAB R2022a software.

### Statistical Analysis

Statistical analysis was performed using GraphPad Prism 8.0.2 statistical software. Two‐tailed Student's *t*‐tests were utilized to assess statistically significant differences between two groups. One‐way ANOVA with Tukey post‐hoc tests were utilized to compare multiple groups and determine statistical significance. Different numbers of asterisks were assigned to the graphs showing the statistical comparison across different groups (ns: non‐significant difference; *: *p* < 0.05; **: *p* < 0.01, ***: *p* < 0.001, ****: *p* < 0.0001).

## Conflict of Interest

The authors declare no conflict of interest.

## Author Contributions

M.K. and S.M.A.M. contributed equally to this work. M.K., S.M.A.M., and S.Y.E. conceived and designed the idea. S.Y.E. supervised all aspects of the project. M.K., S.M.A.M., A.J., S.S., and P.S.P. performed all experiments. M.K., S.M.A.M., and S.Y.E. analyzed the data. M.K., S.M.A.M., and S.Y.E. wrote the draft of the manuscript. All authors discussed and commented on the manuscript.

## Supporting information



Supporting Information

## Data Availability

The data that support the findings of this study are available from the corresponding author upon reasonable request.
