# Supplementary material for: Magnetically Controlled Reversible Photomagnetic Nanoactuators for Dynamic Contrast Enhancement in Optical Coherence Tomography
Source: Adv Mater. 2025 Jun 19;37(35):2419235. doi: 10.1002/adma.202419235 (PMC12412001; doi:10.1002/adma.202419235)
Supplement: Supplementary file 1 — Supporting Information [file ADMA-37-2419235-s001.docx]

**Supporting Information**

**Magnetically controlled reversible photomagnetic nanoactuators for dynamic contrast enhancement in optical coherence tomography**

*Myeongsoo Kim^1,2,4^,* *Samuel M. A. Morais^3,4^, Anamik Jhunjhunwala^1^, Shivashankar Subramanian^1^, Paul S. Pelkowski^1^, and Stanislav Y. Emelianov^1,2,3✉^*

^1^Wallace H. Coulter Department of Biomedical Engineering, Georgia Institute of Technology and Emory University School of Medicine, Atlanta, GA 30332, USA

^2^Petit Institute for Bioengineering and Biosciences, Georgia Institute of Technology, Atlanta, GA 30332, USA

^3^School of Electrical and Computer Engineering, Georgia Institute of Technology, Atlanta, GA, 30332, USA

^4^These authors contributed equally to this work

^✉^ Corresponding Authors: Stanislav. Y. Emelianov ([stas@gatech.edu](mailto:stas@gatech.edu))

**Supplementary Figures**

**
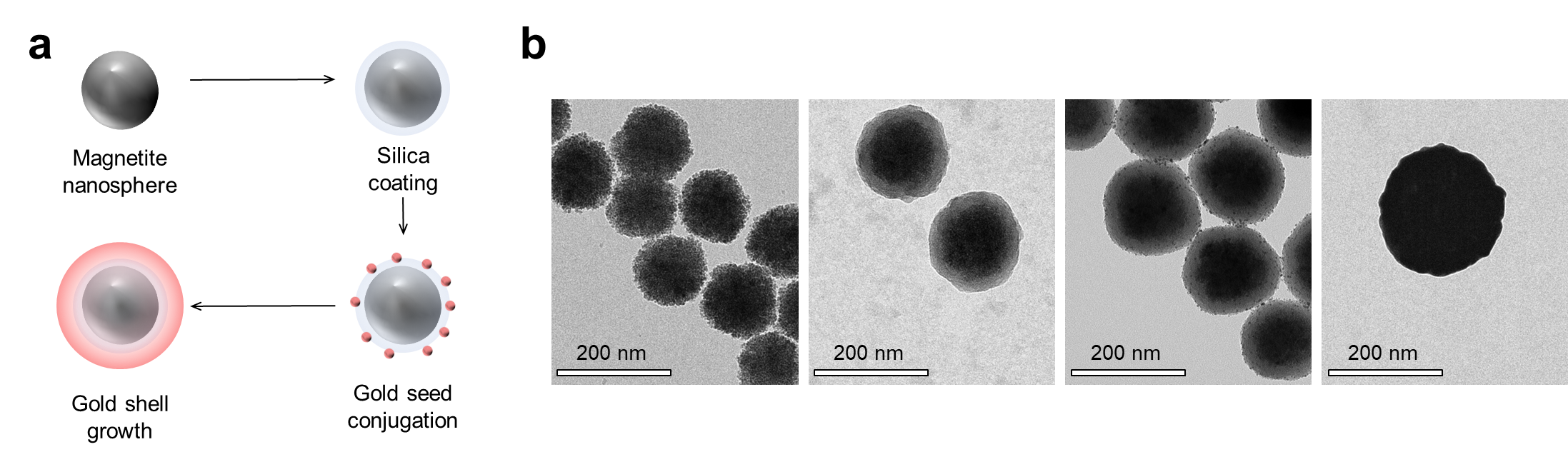
**

**Figure S1.** Synthesis of PMNAs. a) A schematic illustration of the synthetic routes for PMNAs. b) TEM images of Fe_3_O_4_ nanoparticles, Fe_3_O_4_-SiO_2_ nanospheres, gold seed-conjugated Fe_3_O_4_-SiO_2_ nanospheres, and PMNAs, respectively (from left to right).

**
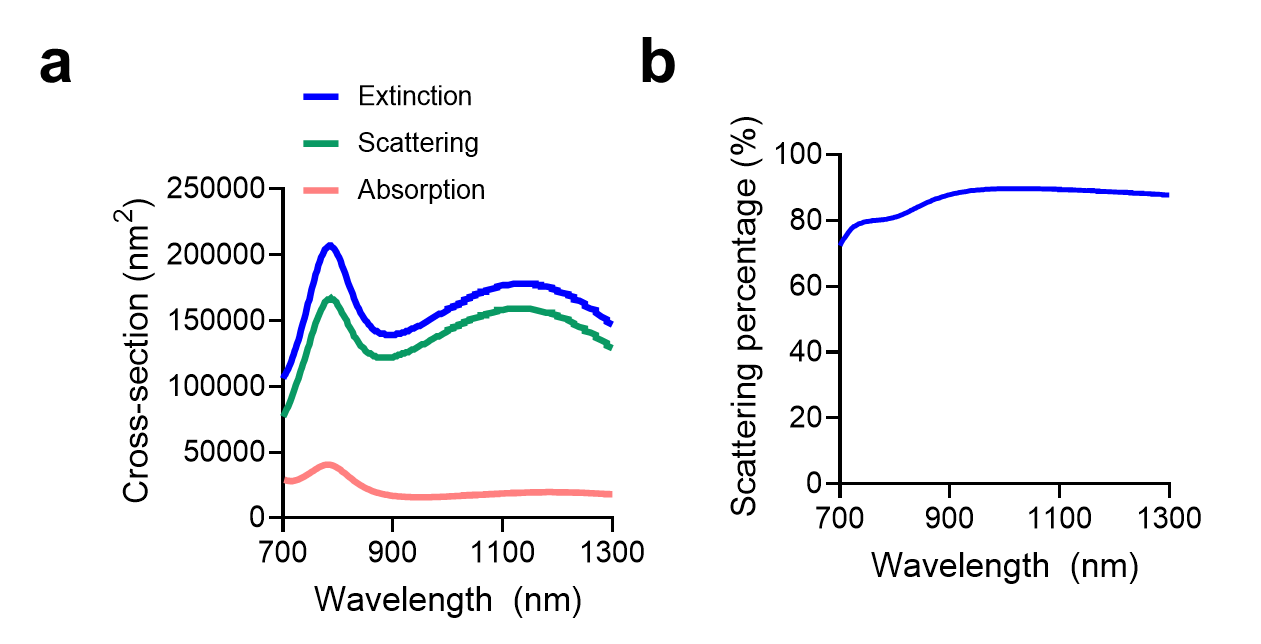
**

**Figure S2.** a) Calculated extinction, scattering, and absorption cross-sections of PMNAs at 700-1300 nm wavelengths. b) Calculated scattering percentages of PMNAs at 700-1300 nm wavelengths.

**
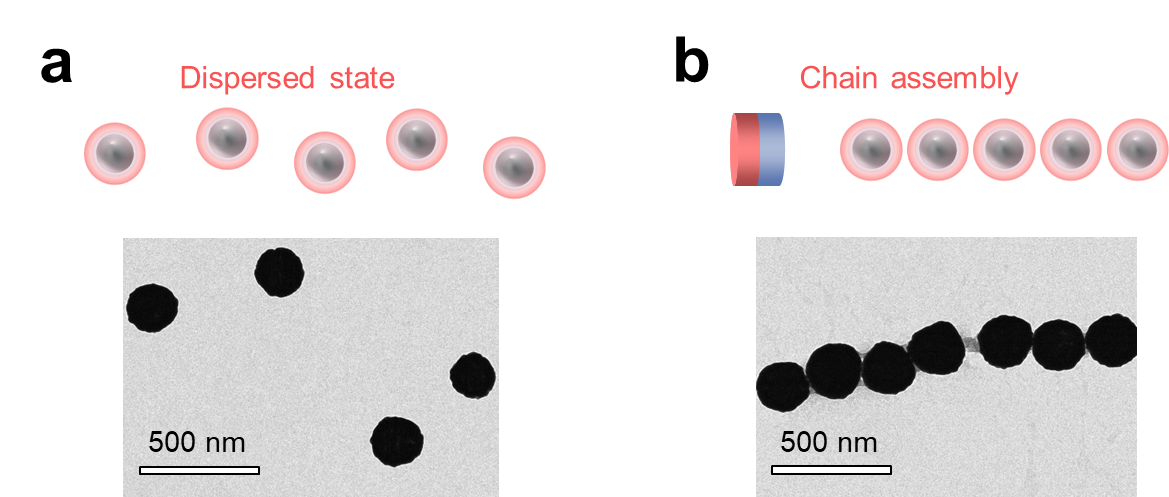
**

**Figure S3.** a, b) TEM images of PMNAs without or with the external magnetic field treatment.

**
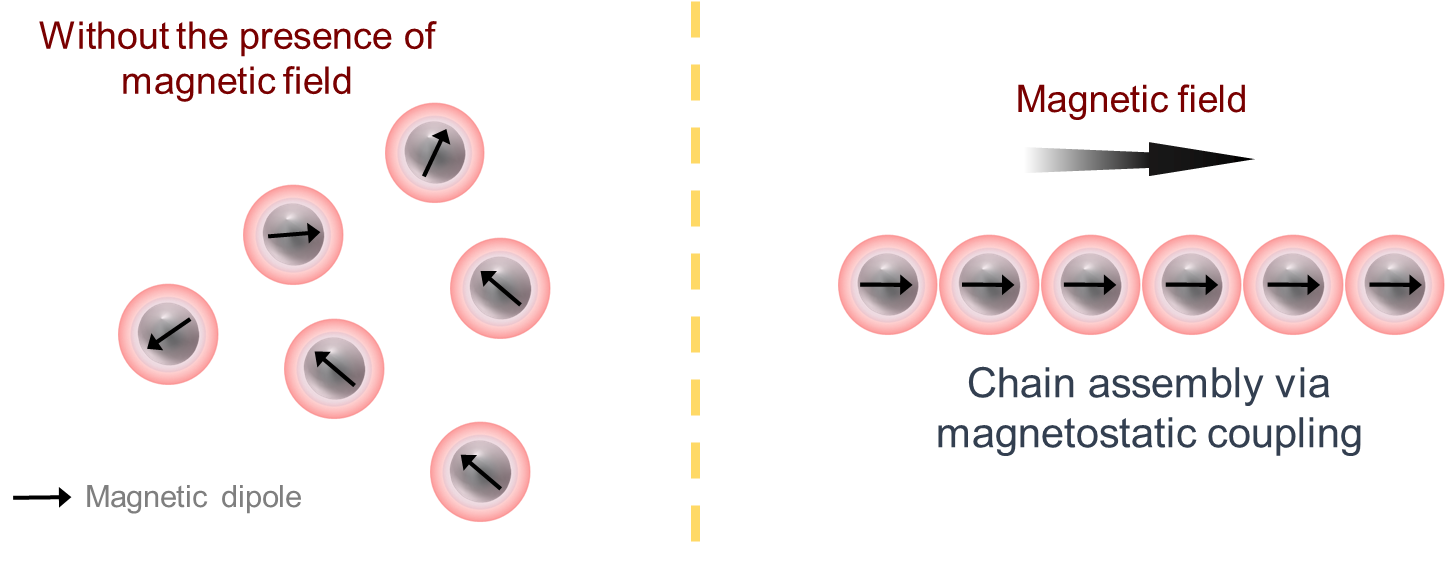
**

**Figure S4.** Schematic illustration of the mechanism for PMNA chain assembly in the presence of an external magnetic field.

**
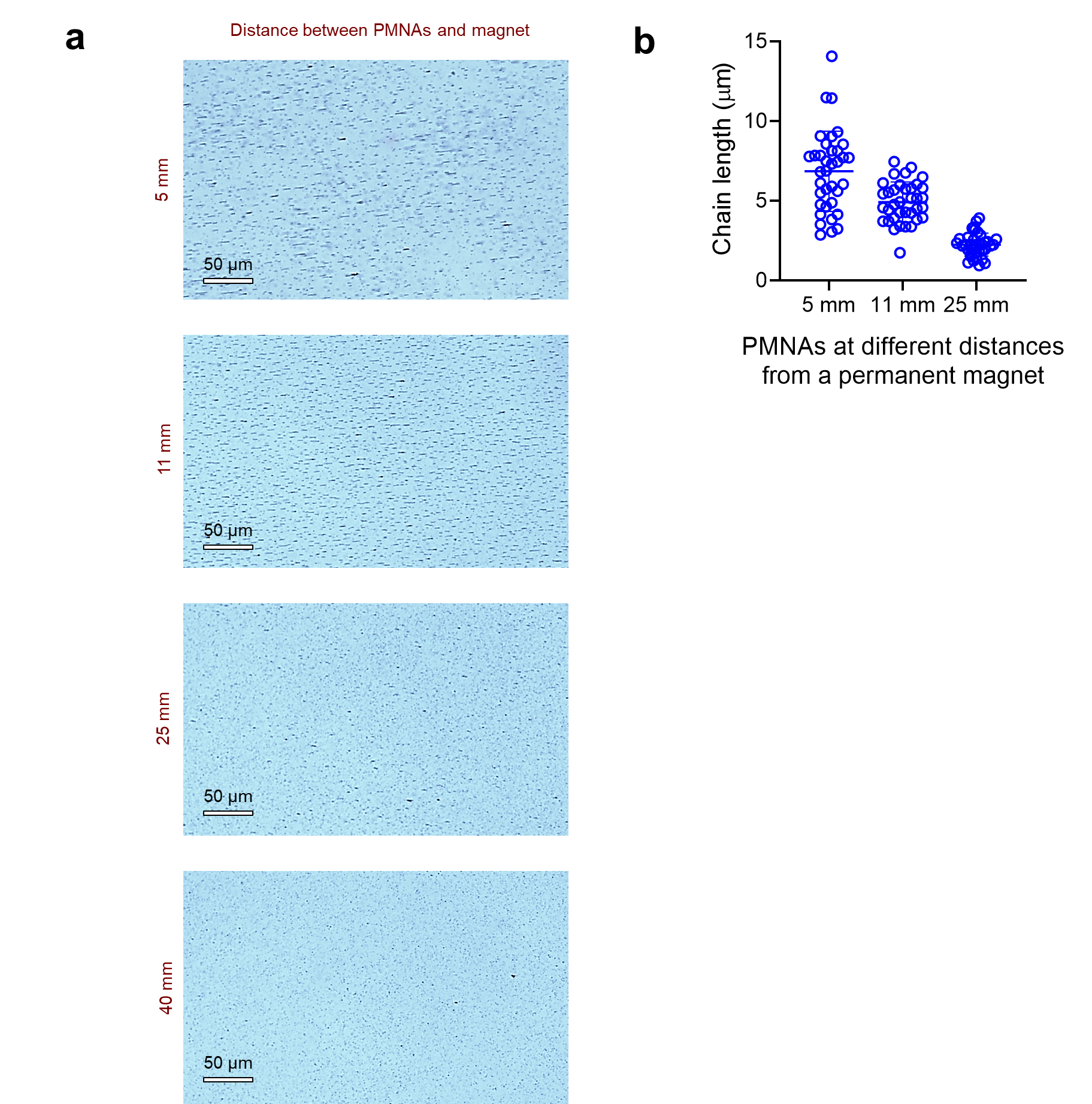
**

**Figure S5.** a) Optical microscope images of PMNAs (160 µg/mL) in water with varying distances from a permanent magnet and b) corresponding chain length distributions (n=36). Data are presented as the mean ± standard deviation. The magnetic field strength applied at the distance of 5 mm from the magnet was approximately 100 mT.

**
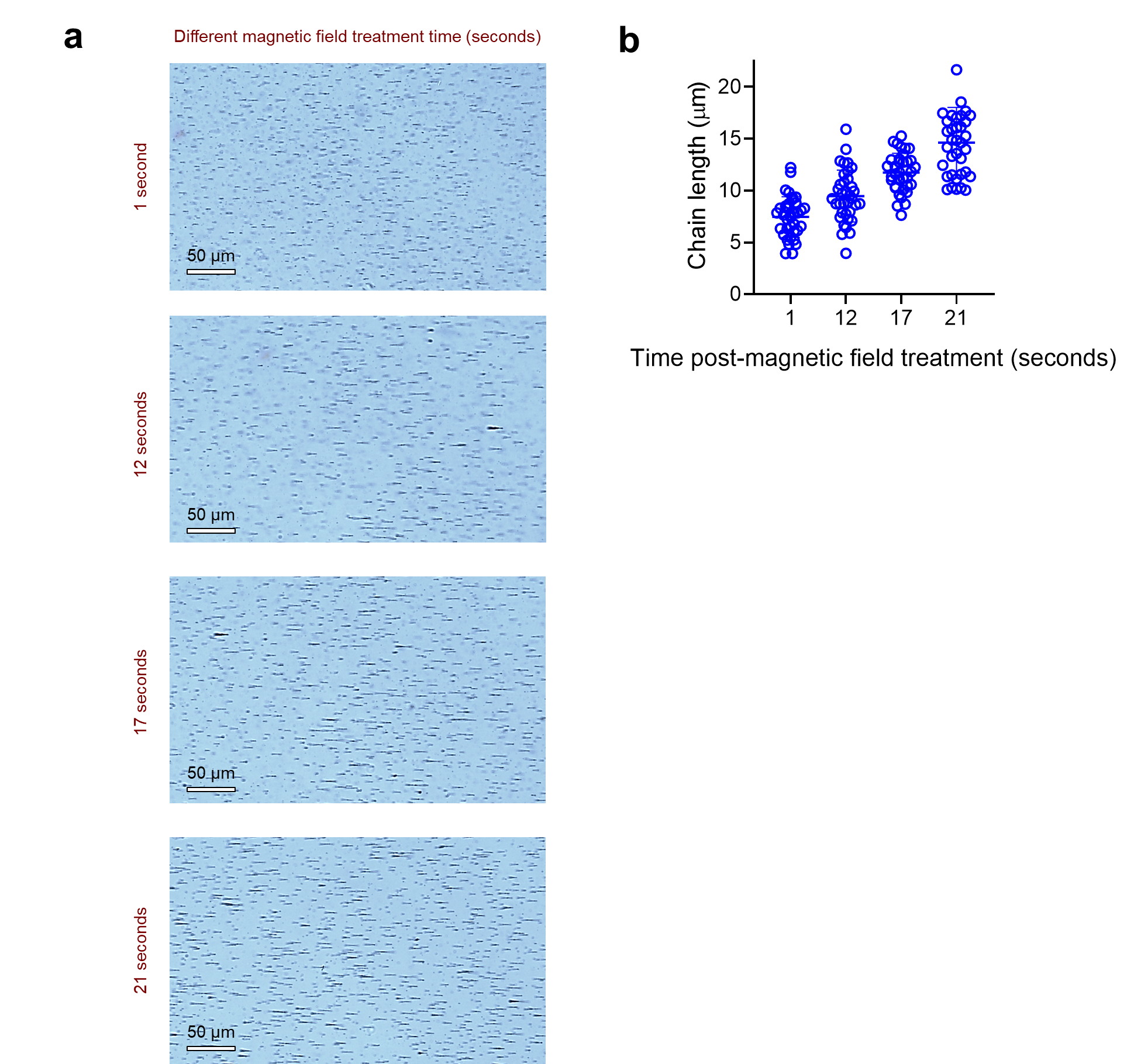
**

**Figure S6.** a) Optical microscope images of PMNAs (160 µg/mL) in water at different time post-magnetic treatment and b) corresponding chain length distributions (n=36). Data are presented as the mean ± standard deviation. The magnetic field strength applied in this experiment was approximately 100 mT.

**
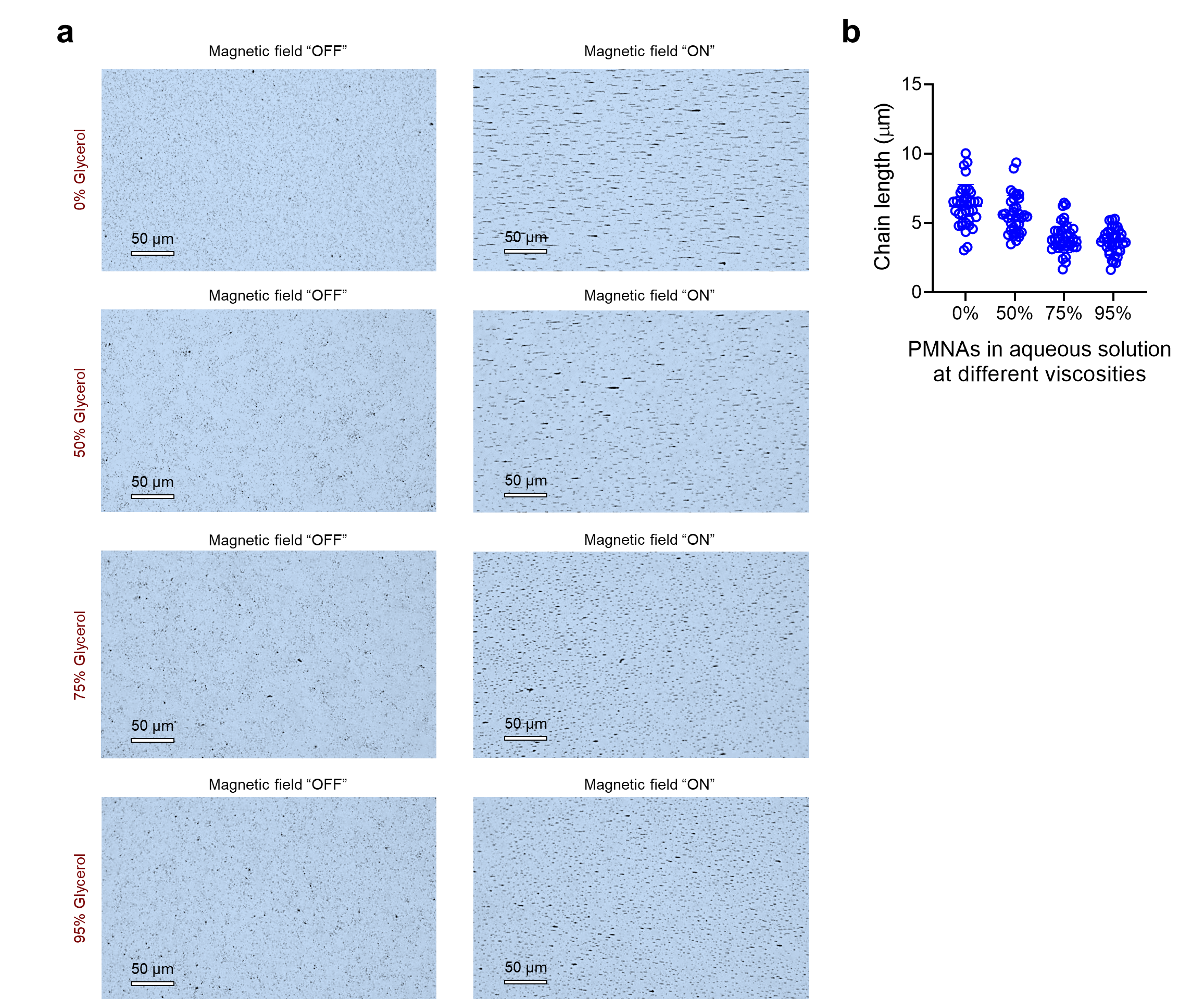
**

**Figure S7.** a) Optical microscope images of PMNAs (160 µg/mL) in glycerol-water mixtures with varying glycerol volume fractions of 0%, 50%, 75%, and 95% upon the modulation of external magnetic field. b) Chain length distributions of PMNAs in glycerol-water mixtures at different glycerol volume fractions upon the exposure to the external magnetic field (n=36). Data are presented as the mean ± standard deviation. The magnetic field strength applied in this experiment was approximately 100 mT.

**
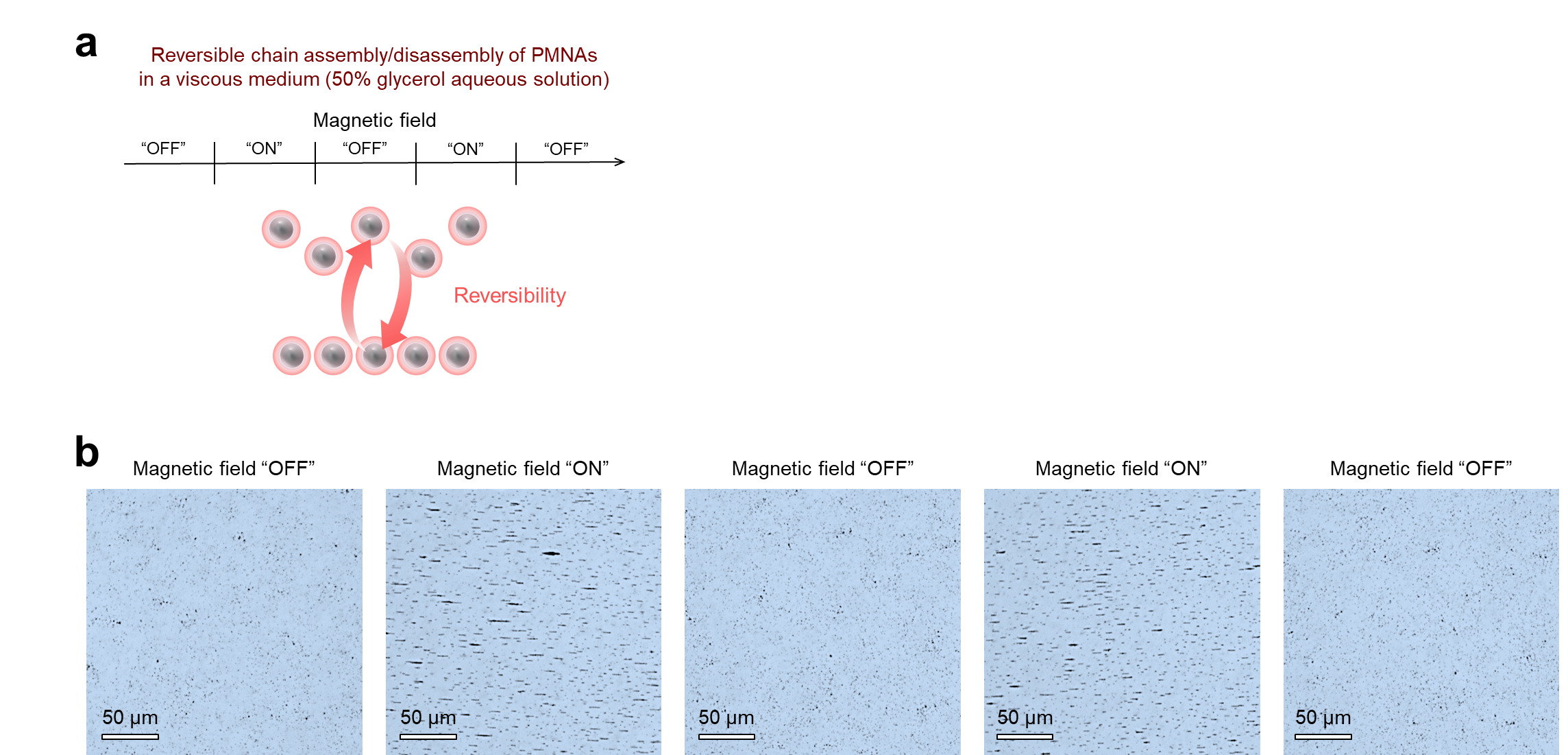
**

**Figure S8.** a, b) Schematic illustration and corresponding optical microscope images of reversible chain assembly and disassembly of PMNAs (160 µg/mL) in 50% glycerol aqueous solution upon the modulation of external magnetic field. The magnetic field strength applied in this experiment was approximately 100 mT.

**
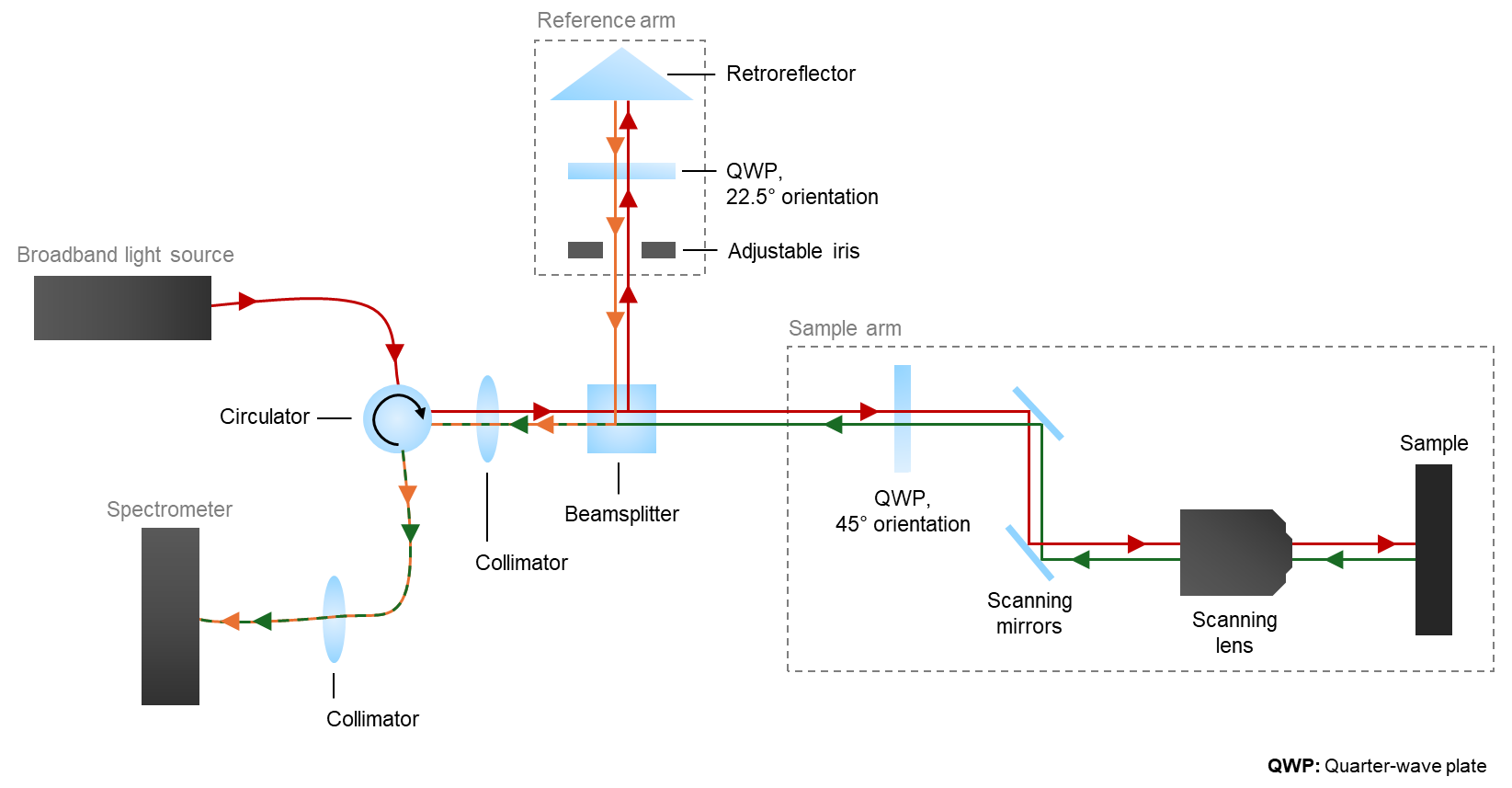
**

**Figure S9.** A schematic illustration of the spectral-domain OCT system.

**
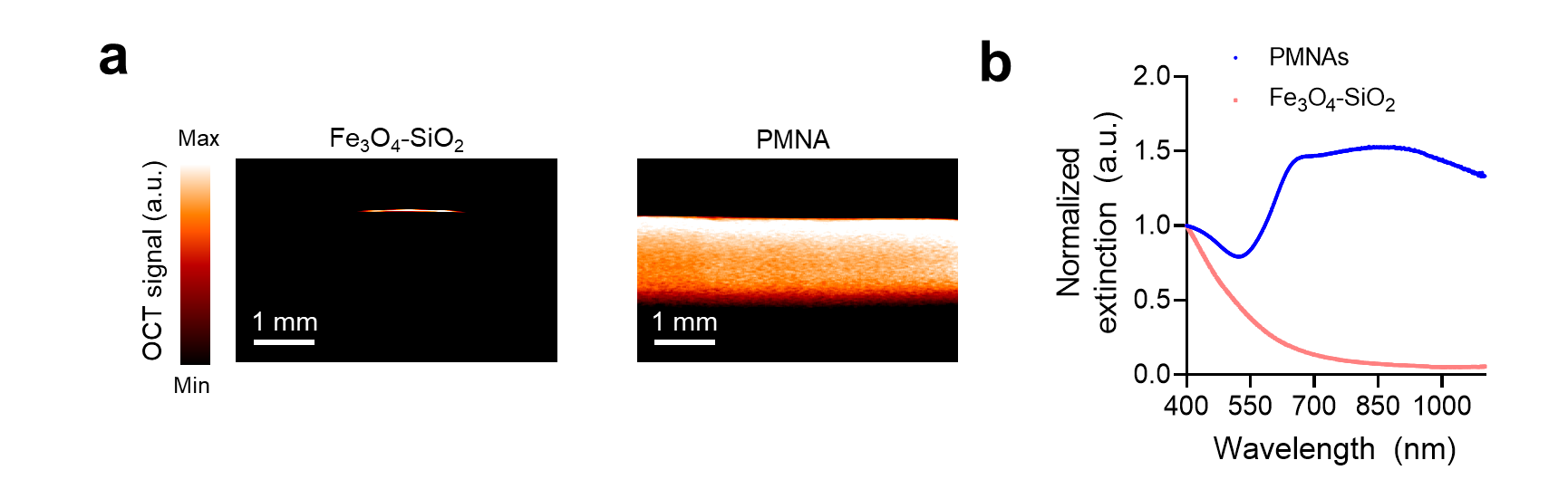
**

**Figure S10.** a) OCT images of Fe_3_O_4_-SiO_2_ nanospheres and PMNAs under light excitation at 1300 nm. b) UV-vis-NIR spectra of Fe_3_O_4_-SiO_2_ nanospheres and PMNAs. The imaging experiments were conducted independently at least three times and similar outcomes were observed.

**Figure S11.** Depth-dependent OCT signal profile of PMNAs in water.

**
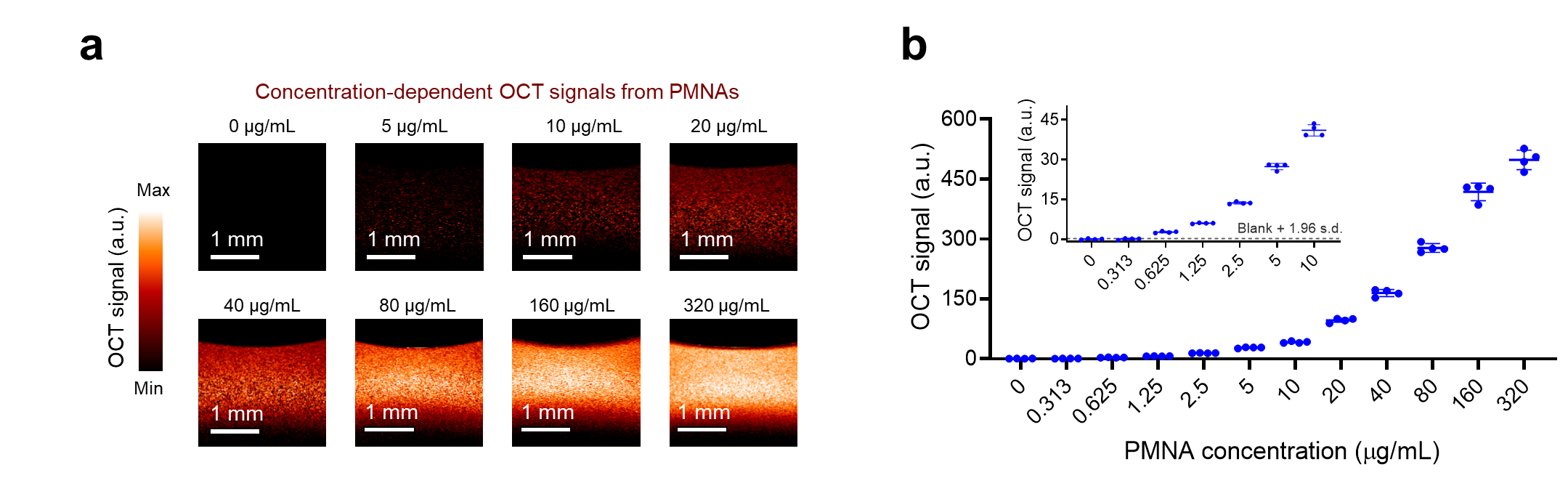
**

**Figure S12.** a, b) OCT signal generation from PMNAs in water at different particle concentrations ranging from 0 µg/mL to 320 µg/mL (n=4). Data are presented as the mean ± standard deviation.

**
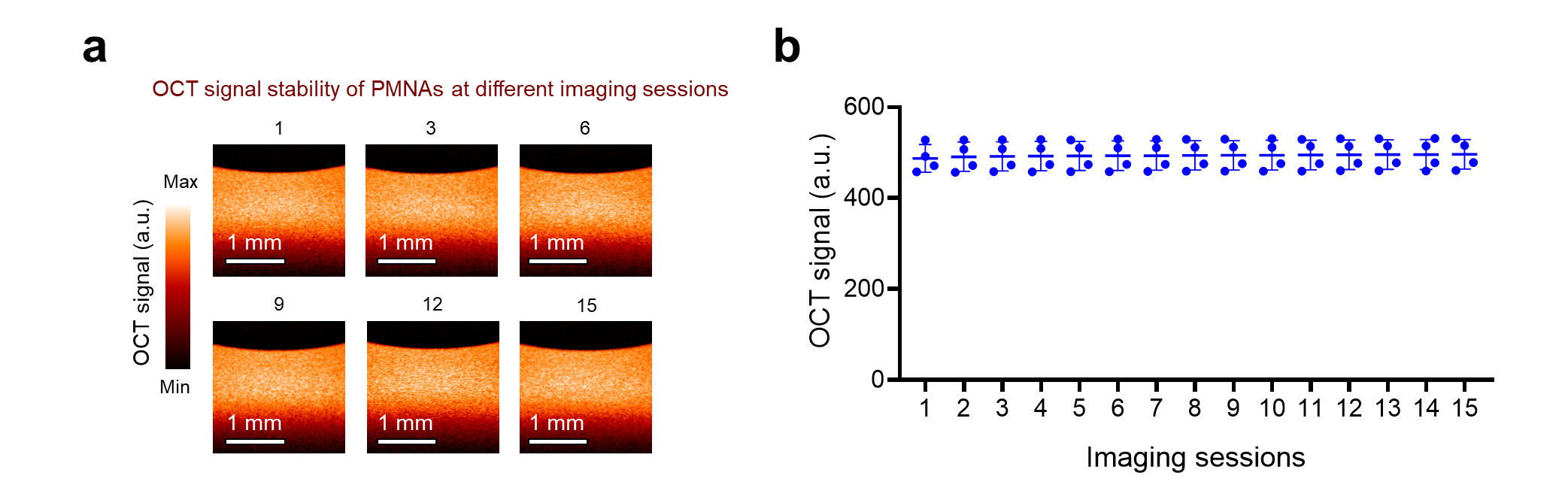
**

**Figure S13.** a, b) OCT signals from PMNAs (320 µg/mL) at different imaging sessions (n=4). Data are presented as the mean ± standard deviation.

*
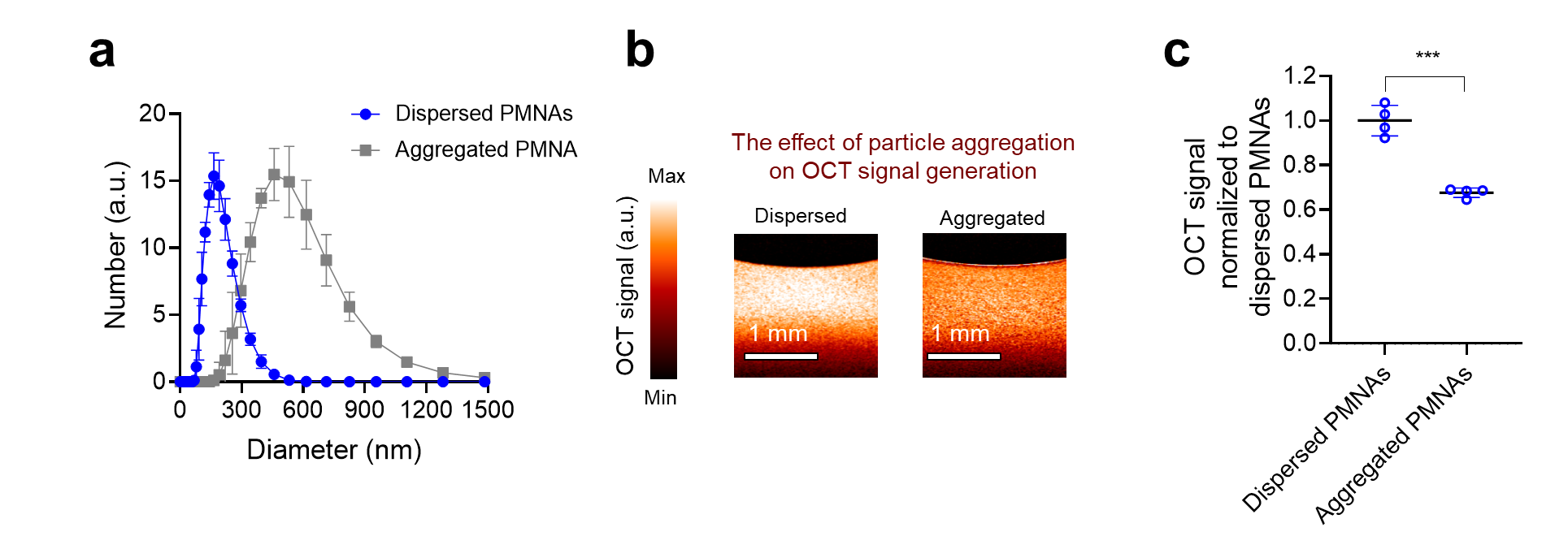
*

**Figure S14.** a) Hydrodynamic diameter distribution of PMNAs (160 µg/mL) in water or 500 mM sodium chloride aqueous solution (n=3). b, c) OCT signal generation from PMNAs in water or 500 mM sodium chloride aqueous solution (n=4). Data are presented as the mean ± standard deviation. The statistical analysis for Figure S14c was conducted using a two-tailed Student’s t-test. The statistically significant difference is represented as the asterisk (***: p < 0.001).

**
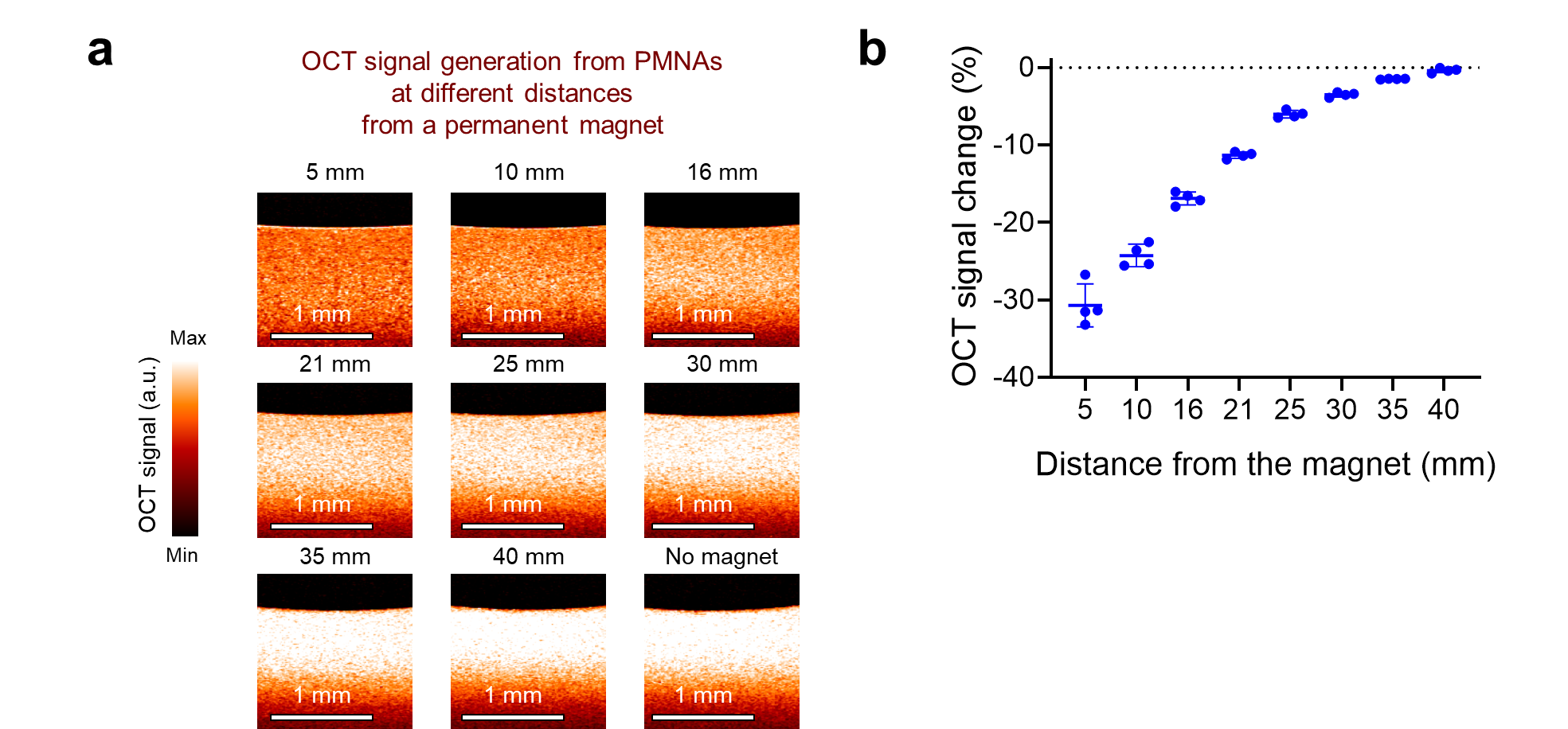
**

**Figure S15.** a, b) OCT signal generation from PMNAs (320 µg/mL) at different distances from the magnet (n=4). Data are presented as the mean ± standard deviation. The magnetic field strength applied at the distance of 5 mm from the magnet was approximately 100 mT. The imaging experiments were repeated independently four times and similar imaging results were obtained.

**
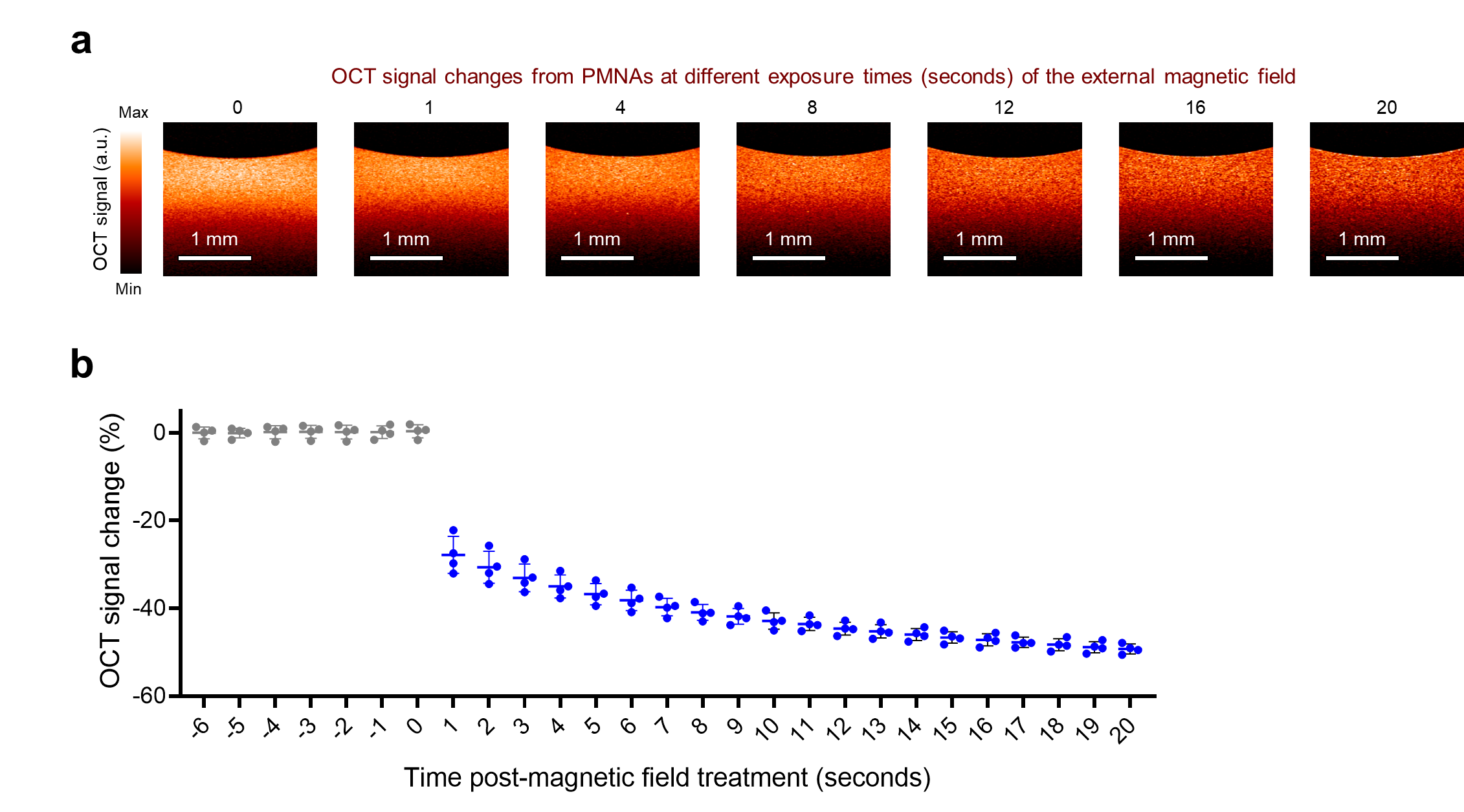
**

**Figure S16.** a, b) OCT signal generation from PMNAs (320 µg/mL) at different time post-magnetic field treatment (n=4). Data are presented as the mean ± standard deviation. The magnetic field strength applied in this experiment was approximately 100 mT. The imaging experiments were repeated independently four times and similar imaging results were obtained.

**
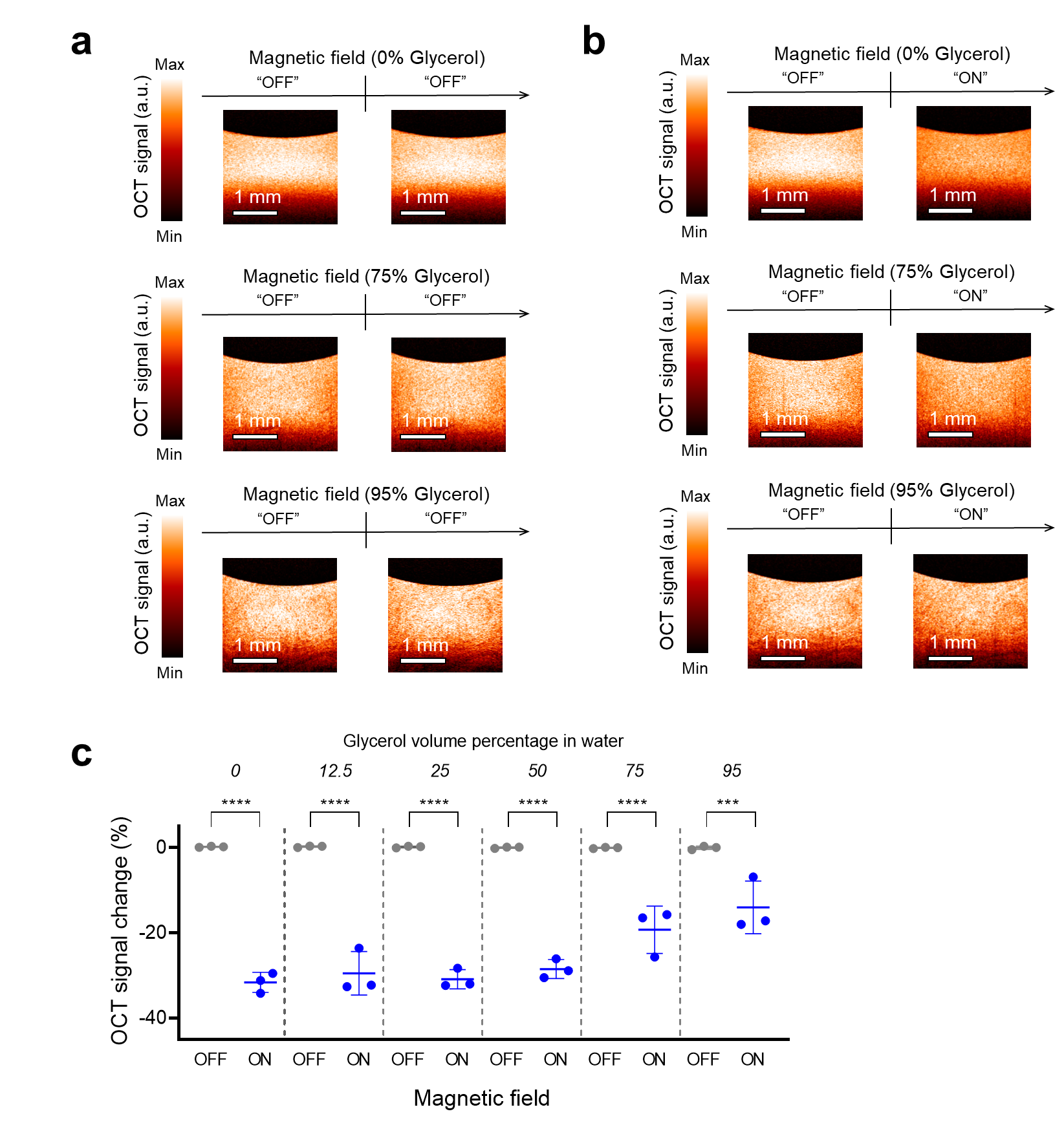
**

**Figure S17.** a-c) Changes in OCT signals from PMNAs (320 µg/mL) in glycerol-water solution at different glycerol volume percentages upon control of the external magnetic field (n=3). Data are presented as the mean ± standard deviation. The statistical analysis was conducted using a one-way ANOVA with Tukey post-hoc test. The statistically significant difference is represented as the asterisk (***: p < 0.001, ****: p < 0.0001). The imaging experiments were repeated independently three times and similar imaging results were obtained.

**
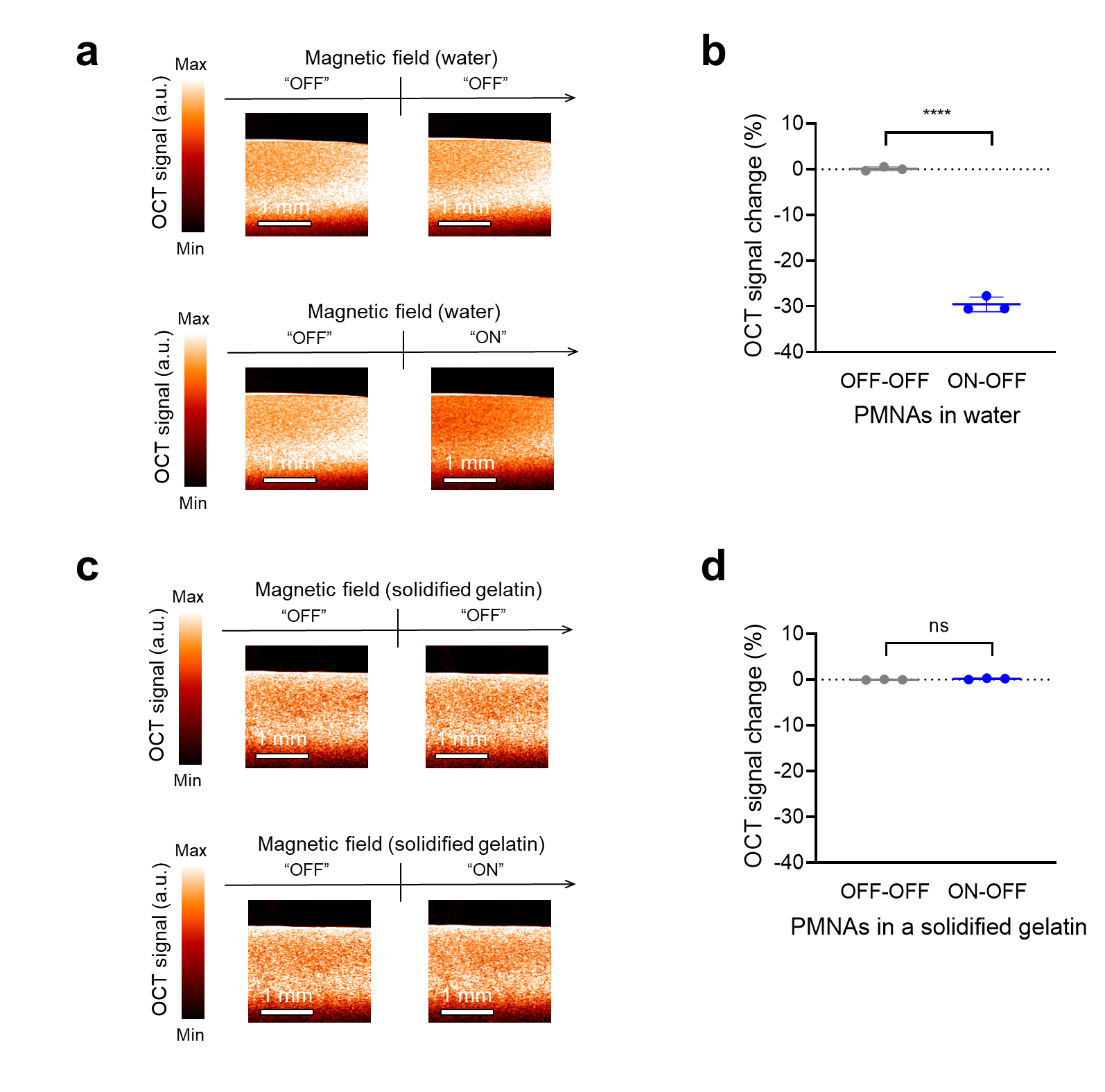
**

**Figure S18.** a, b) Changes in OCT signals from PMNAs (190 µg/mL) in water by modulating the external magnetic field (n=3). c, d) Changes in OCT signals from PMNAs (190 µg/mL) in a solidified gelatin matrix by modulating the external magnetic field (n=3). Data are presented as the mean ± standard deviation. Statistical analyses for Figures S18b and S18d were conducted using a two-tailed Student’s t-test. The statistically significant difference is represented as the asterisk (ns: non-significant, ****: p < 0.0001).

**
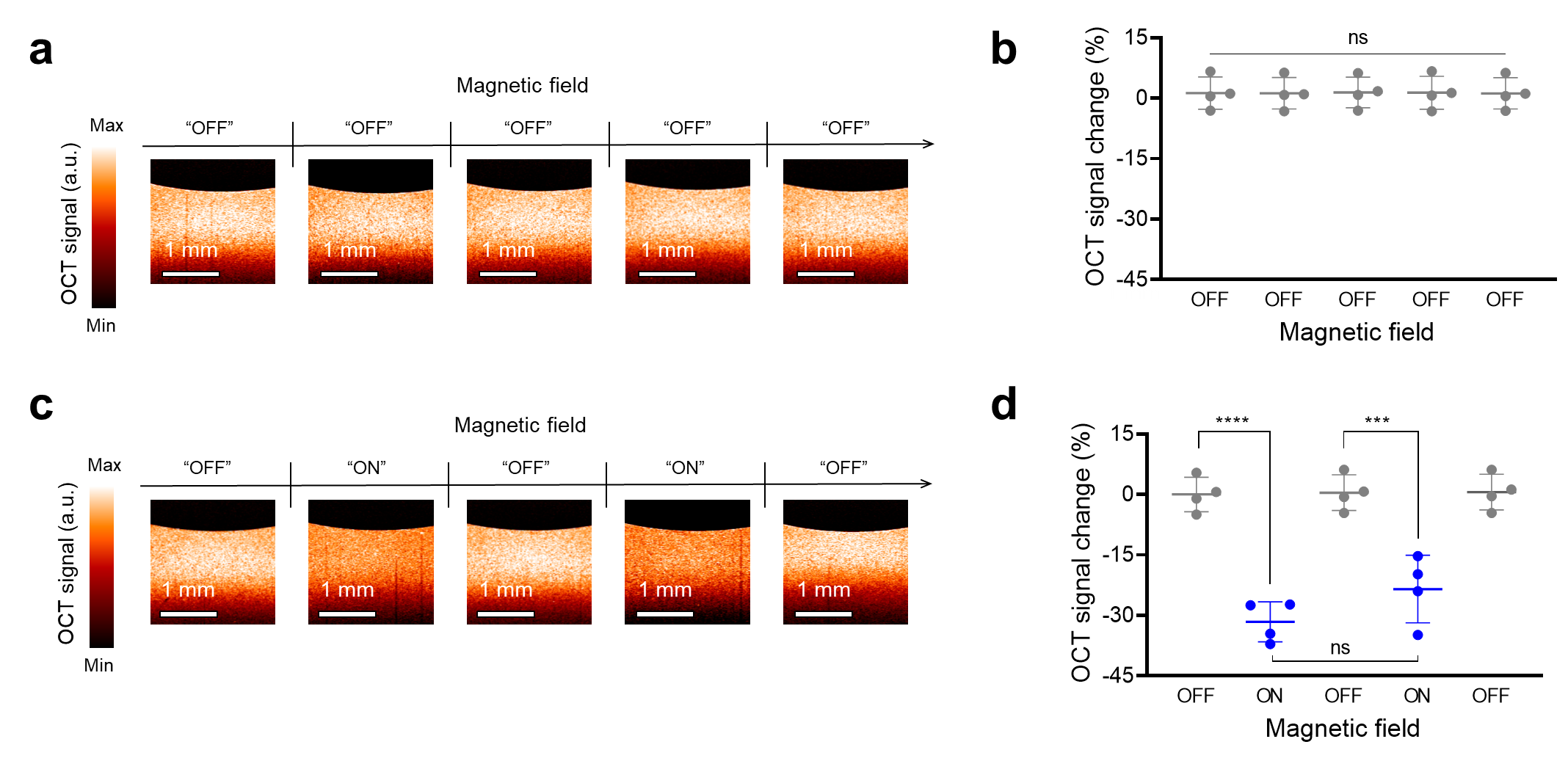
**

**Figure S19.** a-d) Changes in OCT signals from PMNAs (320 µg/mL) in the glycerol water mixture at a glycerol volume fraction of 50% upon modulation of the external magnetic field (n=4). Data are presented as the mean ± standard deviation. The statistical analyses for Figures S19b and S19d were conducted using a one-way ANOVA with Tukey post-hoc test. The statistically significant difference is represented as the asterisk (ns: non-significant, ***: p < 0.001, ****: p < 0.0001). The imaging experiments were repeated independently four times and similar imaging results were obtained.

**
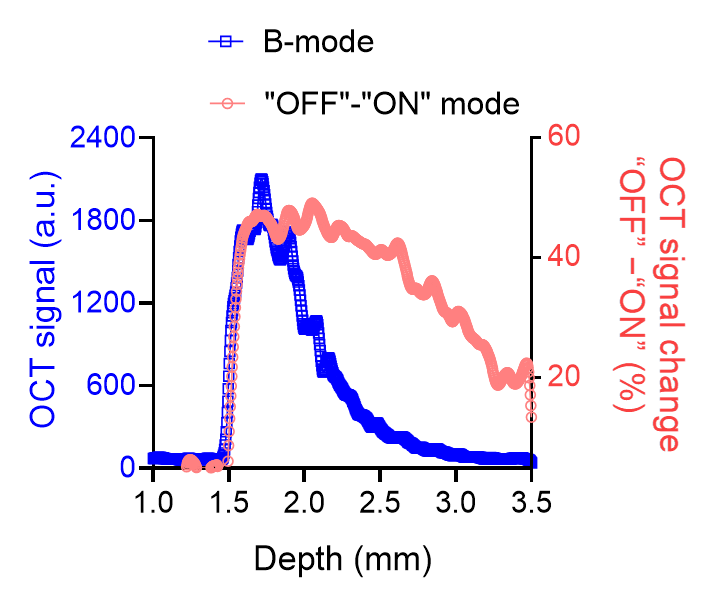
**

**Figure S20.** Depth-dependent profile of original particle OCT signal (B-mode) and the change in OCT signal between “OFF” and “ON” states of PMNAs in water, measured 20 seconds after magnetic field excitation.

**
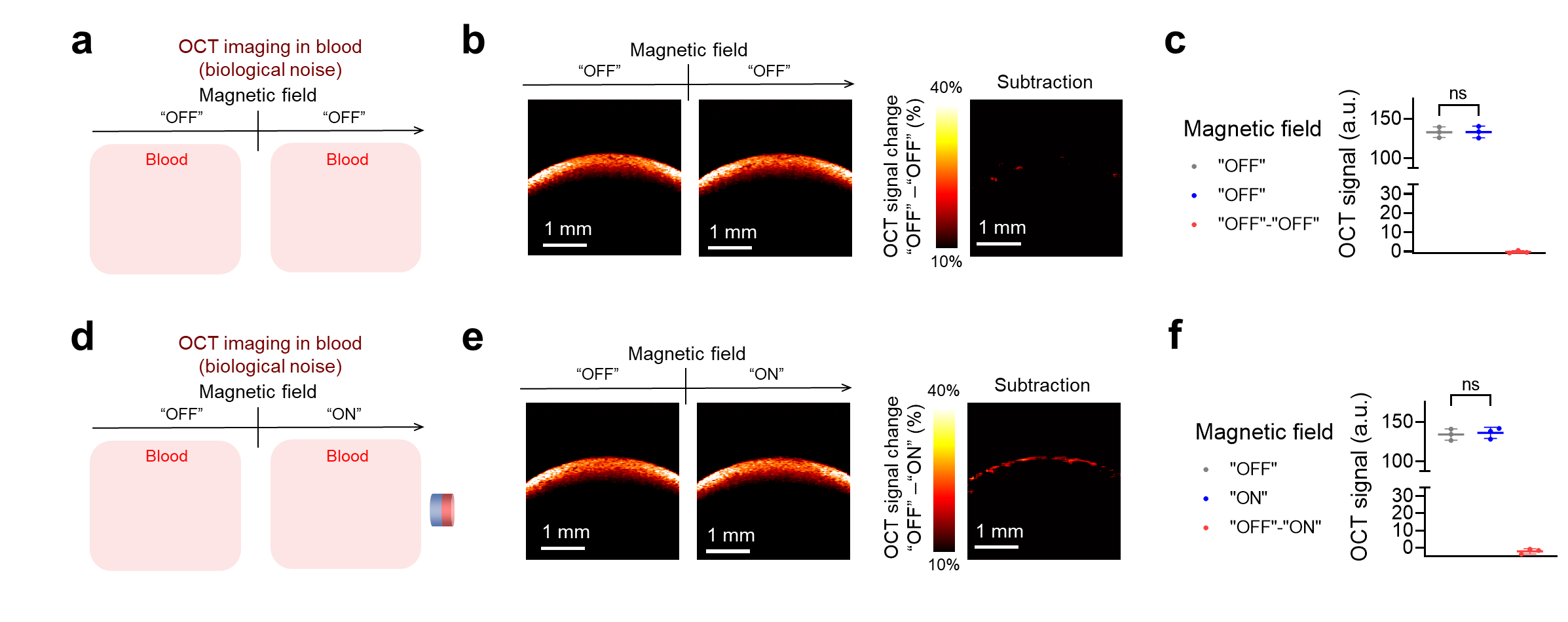
**

**Figure S21.** a-c) OCT signal generation from blood without the modulation of the external magnetic field (n=3). d-f) OCT signal generation from blood with the modulation of the external magnetic field (n=3). Statistical analysis for Figures S21c and S21f was conducted using a two-tailed Student’s t-test. The statistically non-significant difference is represented as ns. The imaging experiments were repeated independently three times and similar imaging results were obtained.

**
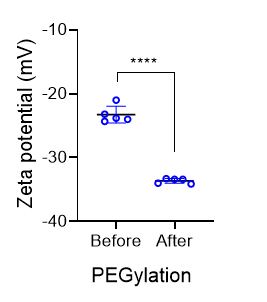
**

**Figure S22.** Zeta potential analysis for characterizing PEGylation of PMNAs (n=5). The statistical analysis was conducted using a two-tailed Student’s t-test. The statistical difference is represented as asterisk (****: p < 0.0001)


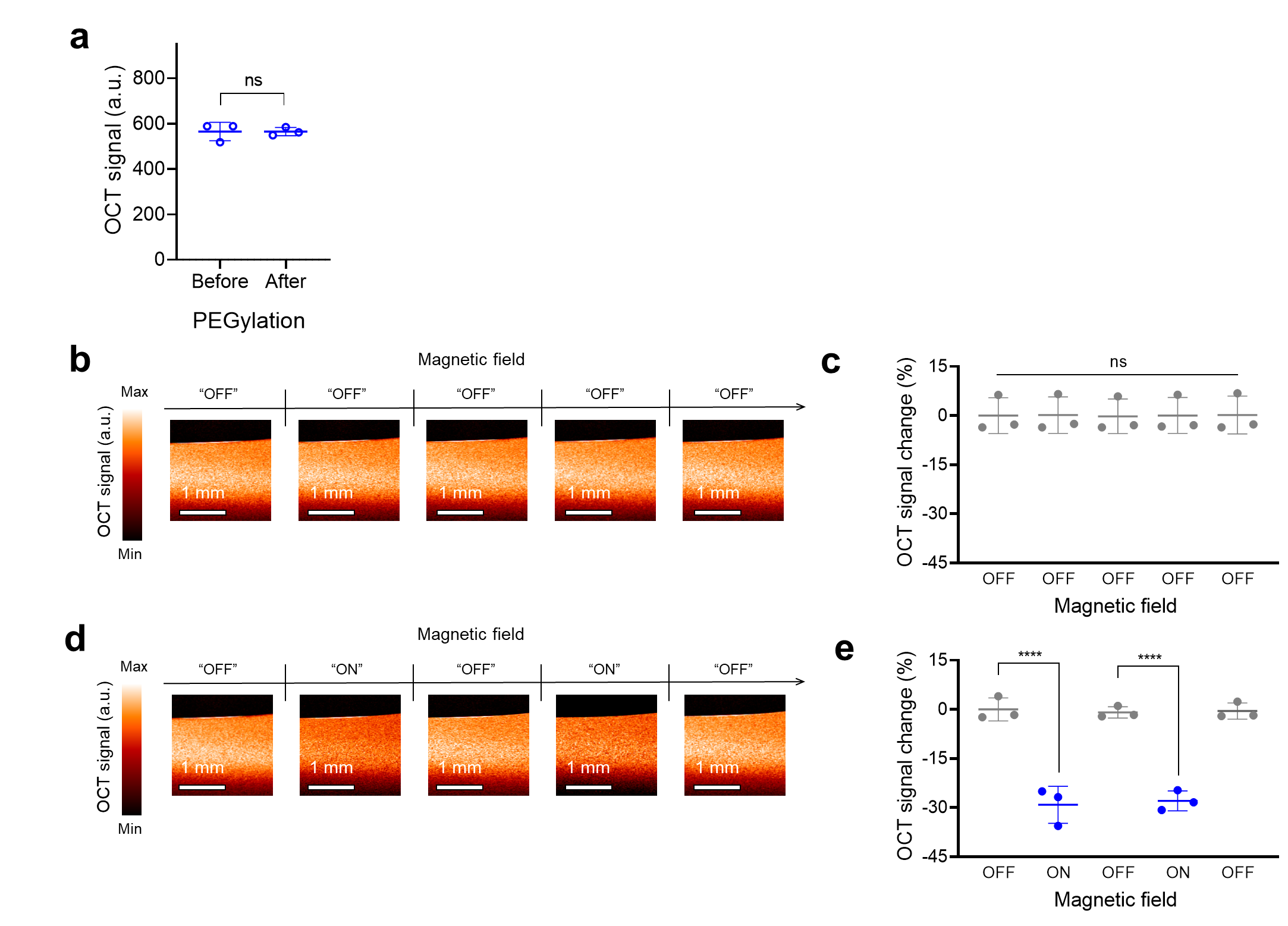


**Figure S23.** a) Comparison of OCT signals from PMNAs (320 µg/mL) before and after PEGylation process (n=3). b-e) Changes in OCT signals from PEGylated PMNAs (320 µg/mL) in water upon control of the external magnetic field (n=3). Data are presented as the mean ± standard deviation. The statistical analysis for Figure S23a was conducted using a two-tailed Student’s t-test. The statistical analyses for Figures S23c and S23e were conducted using a one-way ANOVA with Tukey post-hoc test. The statistically significant difference is represented as the asterisk (ns: non-significant, ****: p < 0.0001). The imaging experiments were repeated independently three times and similar imaging results were obtained.

**Figure S24.** a) OCT signal generation from PMNAs (300 µg/mL) at different pH conditions (n=5). Data are presented as the mean ± standard deviation. The statistical analysis was conducted using a one-way ANOVA with Tukey post-hoc test. The statistically non-significant difference is represented as ns.

**
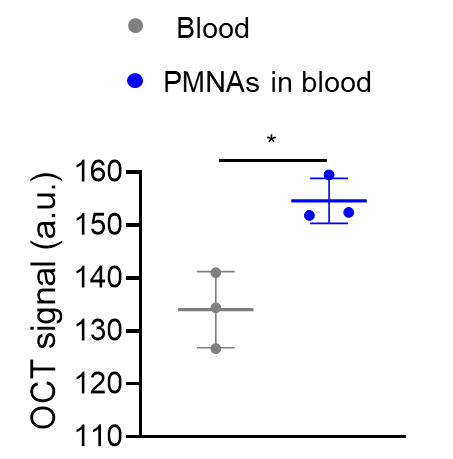
**

**Figure S25.** A comparison of OCT signals between blood and PMNA-dispersed blood (n=3). PMNA concentration was 213 µg/mL. The statistical analysis was conducted using a two-tailed Student’s t-test. The statistical difference is represented as asterisk (*: p < 0.05).

**
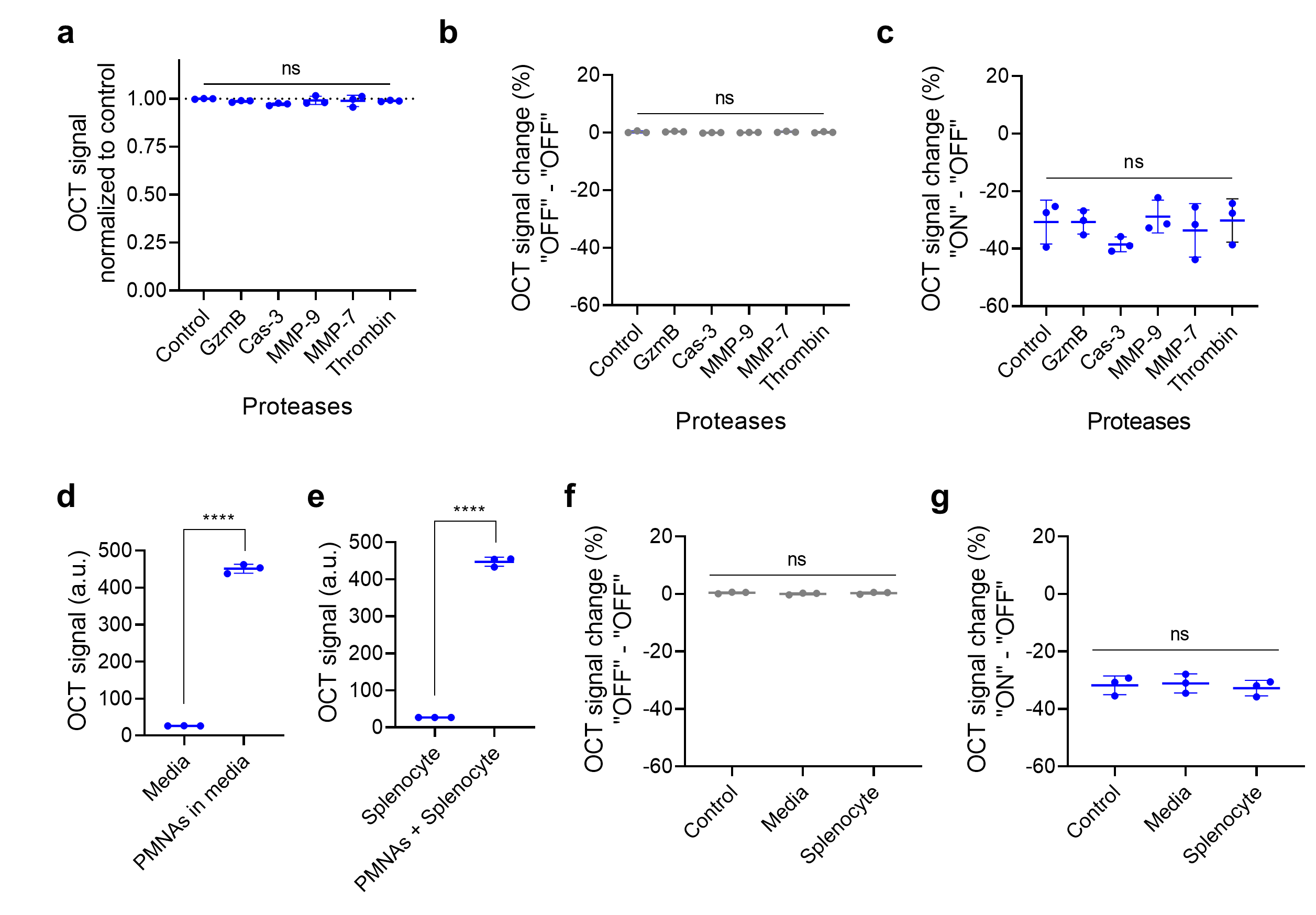
**

**Figure S26.** a) OCT signal generation from PMNAs (280 µg/mL) in the presence of different proteases (n=3). b, c) Changes in OCT signals from PMNAs (280 µg/mL) in the presence of 17 nM different proteases by modulating the external magnetic field (n=3). d, e) OCT signal generation from PMNAs in cell culture medium or cell culture medium containing mouse splenocytes (n=3). f, g) Changes in OCT signals from PMNAs (174 µg/mL) in cell culture medium or cell culture medium containing the mouse splenocytes with cell density of 1M cells/mL (n=3). Data are presented as the mean ± standard deviation. The statistical analyses for Figures S26a, S26b, S26c, S26f, and S26g were conducted using a one-way ANOVA with Tukey post-hoc test. The statistical analyses for Figures S26d and S26e were conducted using a two-tailed Student’s t-test. The statistically significant difference is represented as the asterisk (ns: non-significant, *: p < 0.05, ****: p < 0.0001).

**
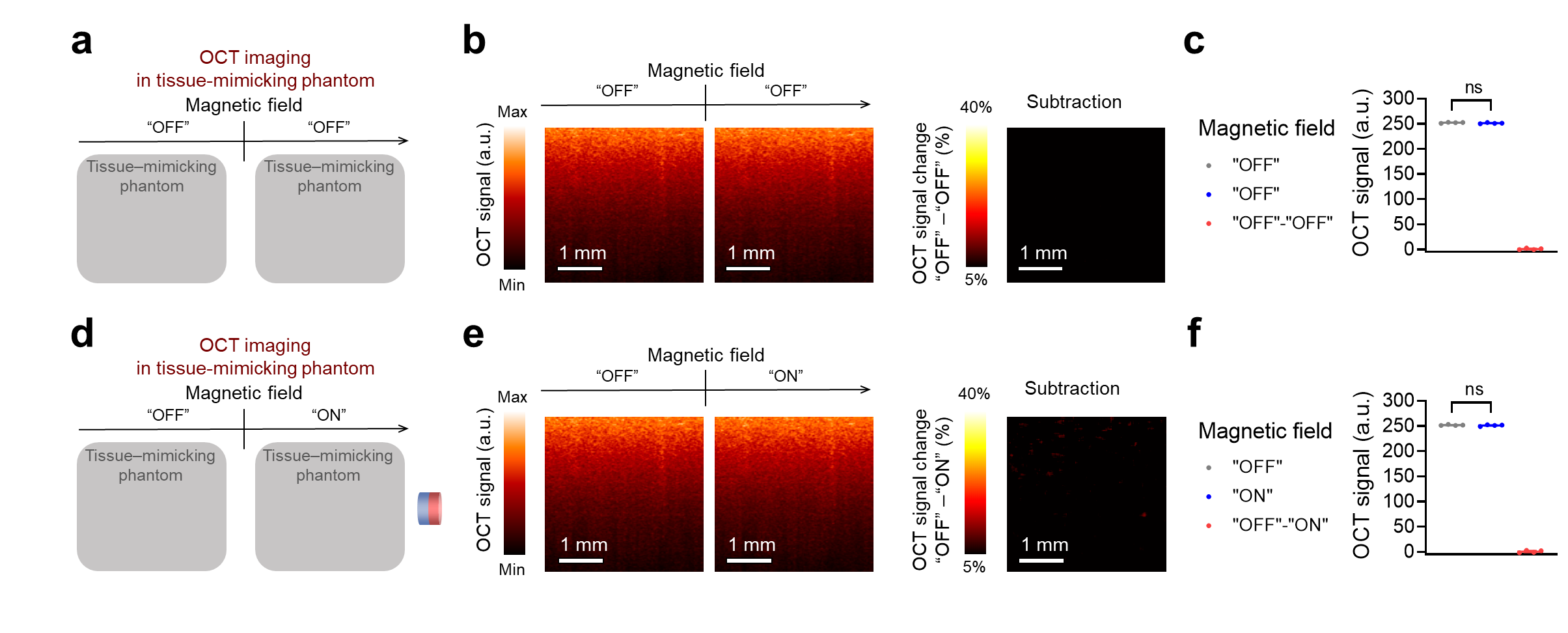
**

**Figure S27.** a-c) OCT signal generation from the tissue-mimicking phantom without the modulation of the external magnetic field (n=4). d-f) OCT signal generation from the tissue-mimicking phantom with the modulation of the external magnetic field (n=4). The statistical analysis for Figures S27c and S27f was conducted using a two-tailed Student’s t-test. The statistically non-significant difference is represented as ns. The imaging experiments were repeated independently four times and similar imaging results were obtained.


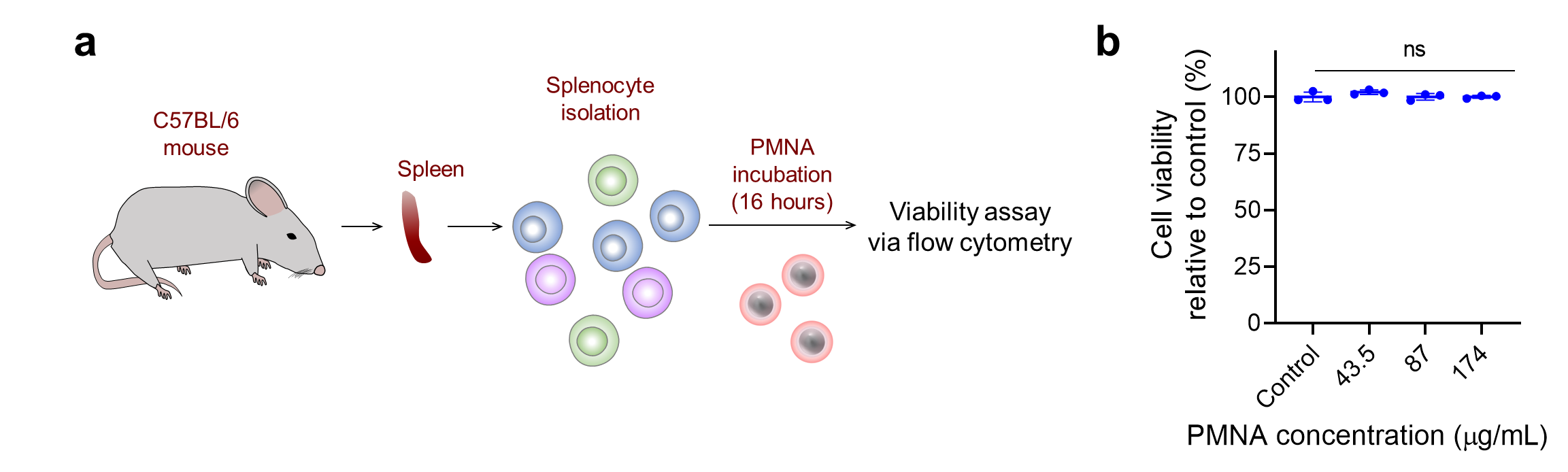


**Figure S28.** a) Schematic illustration of the experiment to investigate the PMNA cytotoxicity against mouse splenocytes. b) Cell viability of mouse splenocytes at different concentrations of PMNAs (n=3). The statistical analysis was conducted using a one-way ANOVA with Tukey post-hoc test. The statistically non-significant difference is represented as ns.

**
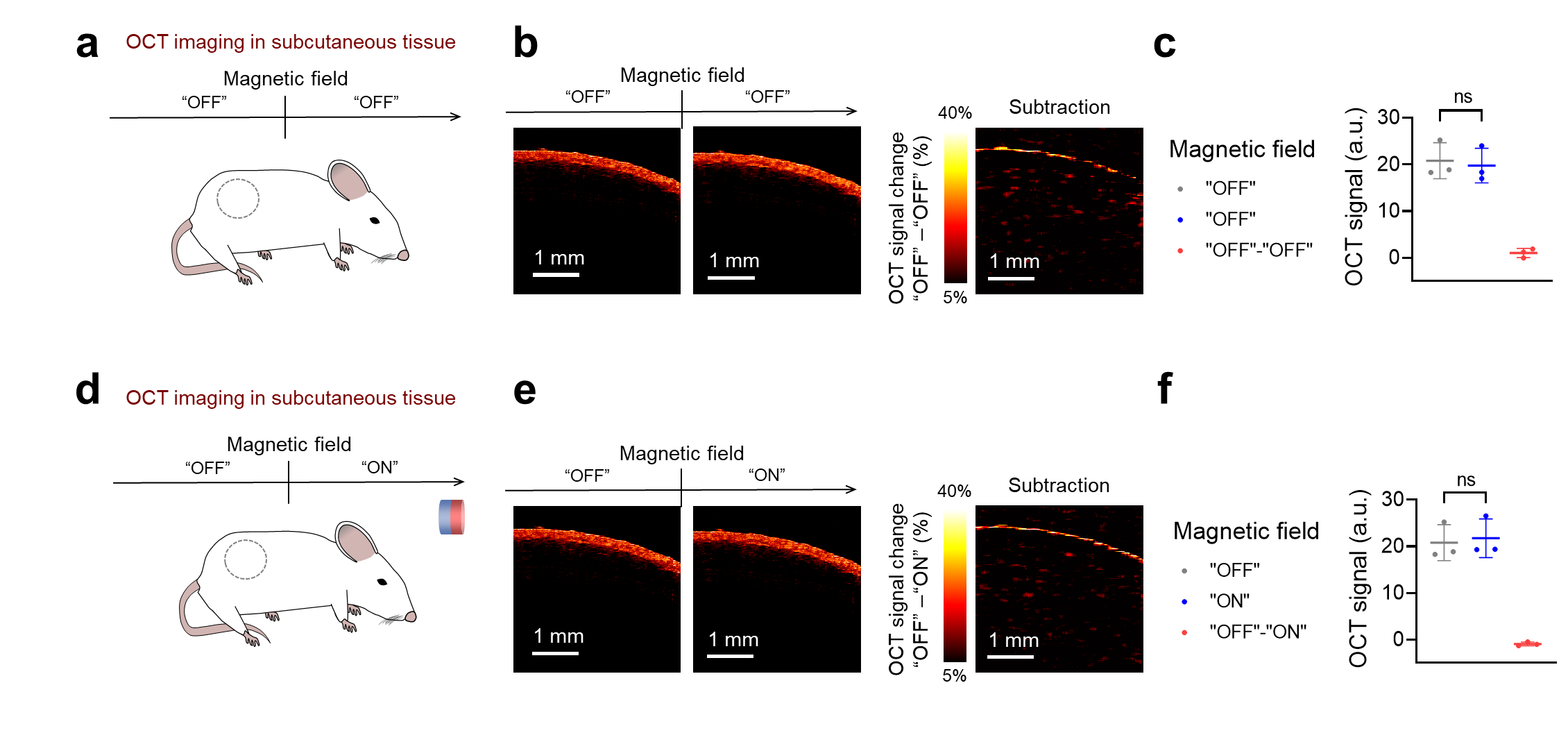
Figure S29.** a-c) OCT signal generation from the OCT signal generation from the mouse tissue without the modulation of the external magnetic field (n=3). d-f) OCT signal generation from the OCT signal generation from the mouse tissue with the modulation of the external magnetic field (n=3). The statistical analysis for Figures S29c and S29f was conducted using a two-tailed Student’s t-test. The statistically non-significant difference is represented as ns. The imaging experiments were repeated independently three times and similar imaging results were obtained.

**
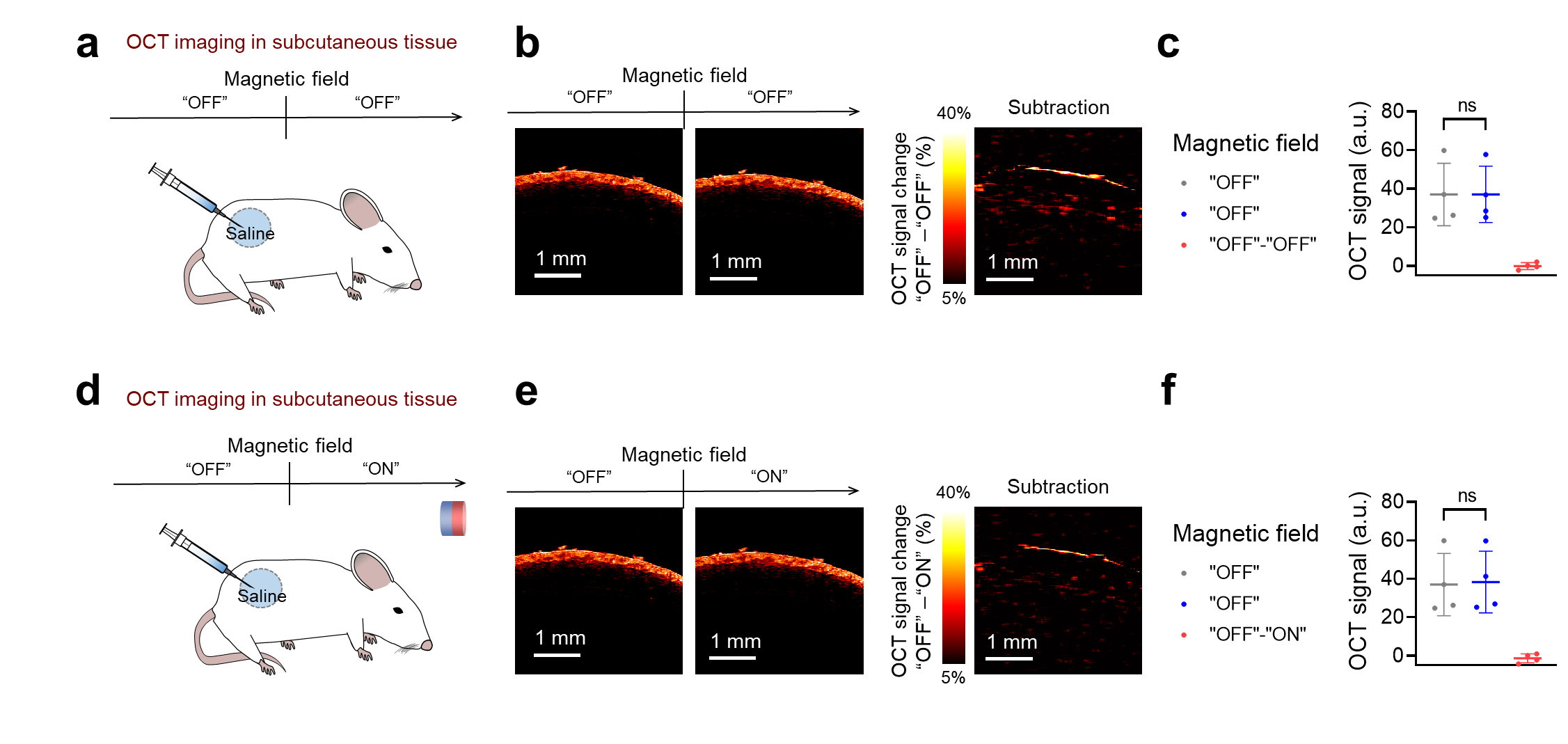
**

**Figure S30.** a-c) OCT signal generation from the OCT signal generation from saline solution in the mouse tissue after subcutaneous solution injection without the modulation of the external magnetic field (n=4). d-f) OCT signal generation from the OCT signal generation from saline solution in the mouse tissue after subcutaneous solution injection with the modulation of the external magnetic field (n=4). The statistical analysis for Figures S30c and S30f was conducted using a two-tailed Student’s t-test. The statistically non-significant difference is represented as ns. The imaging experiments were repeated independently four times and similar imaging results were obtained.

**
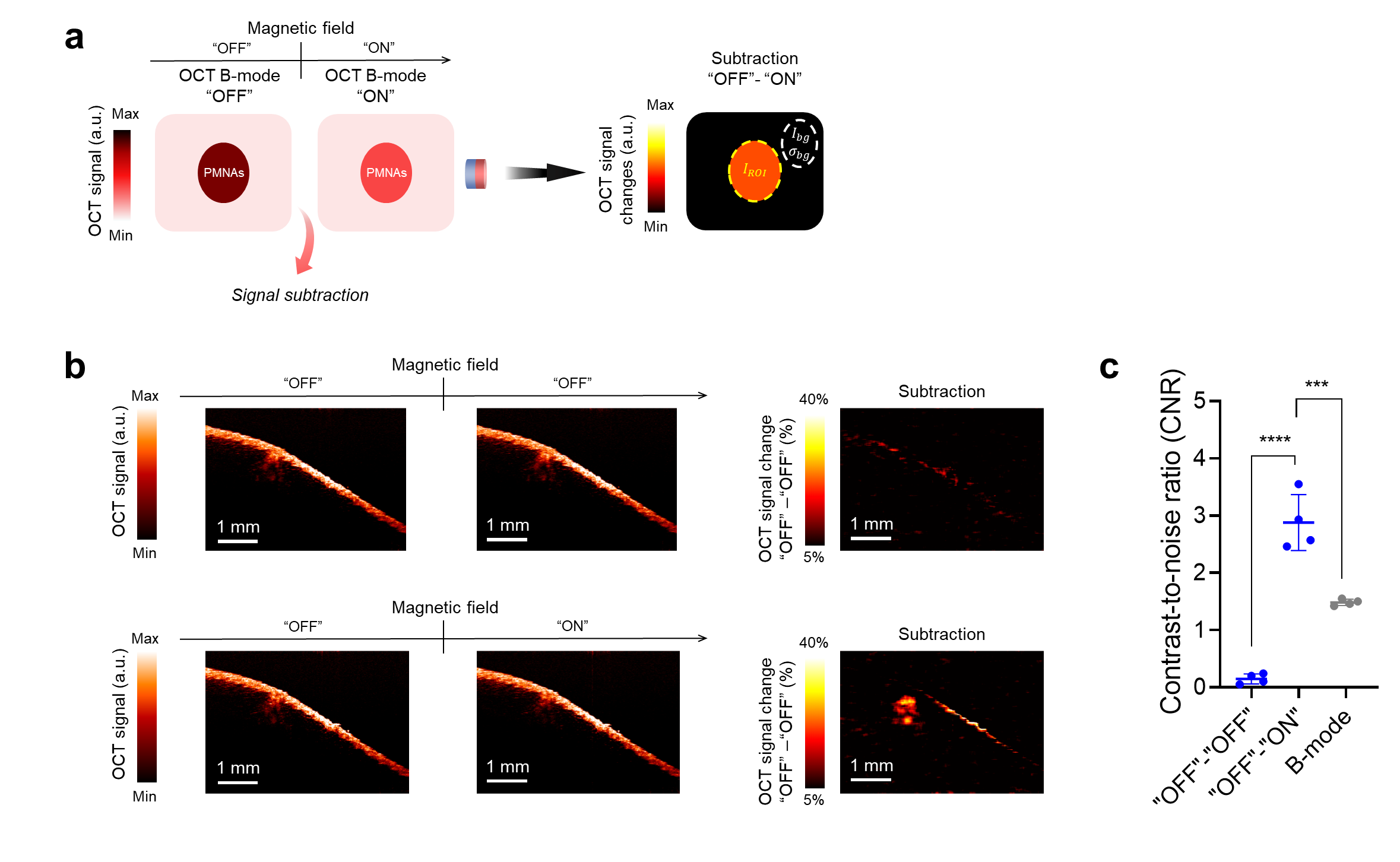
**

**Figure S31.** a) Schematic of CNR calculation from the differential “OFF”-“ON” mode images. Based on OCT B-mode imaging, a region of interest (ROI) was defined around the particle injection area, and the mean OCT signal intensity within this region was calculated (I_ROI_). Another ROI was selected in a background area to determine the mean background intensity (I_bg_) and the standard deviation of the noise (σ_bg_). b, c) OCT imaging of PMNAs (20 µg) in the mouse tissue after subcutaneous particle injection with the modulation of external magnetic field, along with the corresponding CNR (n=4). Data are presented as the mean ± standard deviation. The statistical analysis was conducted using a one-way ANOVA with Tukey post-hoc test. The statistically significant difference is represented as the asterisk (***: p < 0.001, ****: p < 0.0001). The imaging experiments were repeated independently four times and similar imaging results were obtained.


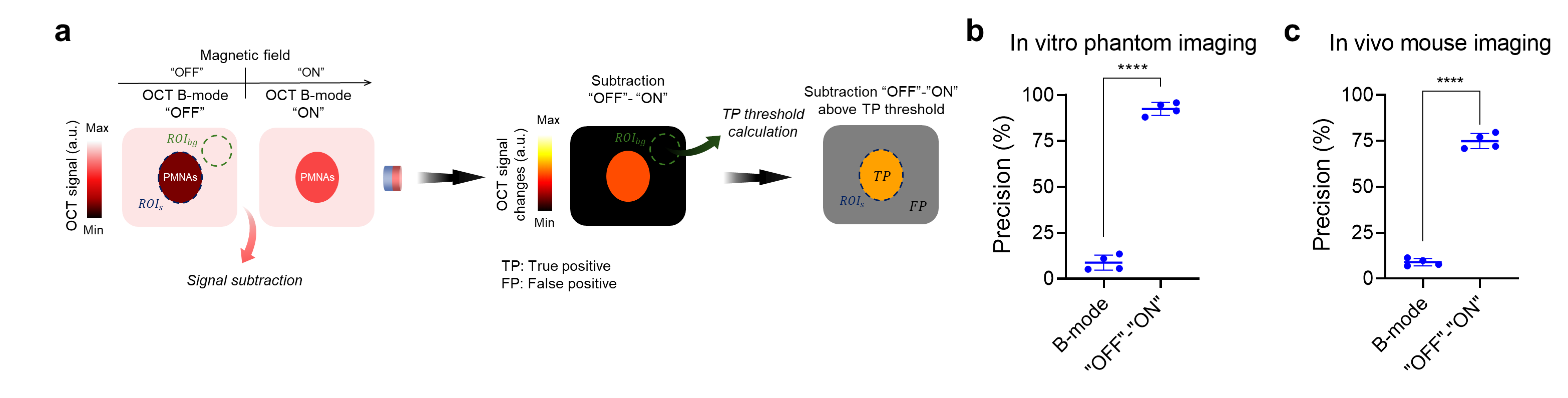


**Figure S32.** a) Schematic of precision calculation from the differential “OFF”-“ON” images. ROI_s_ and ROI_bg_ refer to the regions of interest defined around the particle injection area and the background, respectively. TP corresponds to the true positive values (the correctly identified particle-associated signals) and FP to false positives (the background signals incorrectly classified as particle signals). b, c) Precision of particle-associated signal detection from B-mode and differential images in vitro phantom imaging (n=4) and in vivo mouse imaging (n=4). Data are presented as the mean ± standard deviation. The statistical analyses were conducted using a two-tailed Student’s t-test. The statistically significant difference is represented as the asterisk (****: p < 0.0001).

For the in vitro experiments, a phantom with tube-shaped inclusion was used to confine PMNAs within a well-defined region, thereby serving as a reliable ground truth for classifying true positives and false positives pixels. However, for the in vivo setting, a definitive ground truth is inherently difficult to obtain due to potential dispersion of particles after injection. In this case, we used the injection site that was identified via B-mode imaging as a proxy for the region of true positives. This method may be a conservative estimation, as some signals outside the injection site might correspond to dispersed particles and thus true positives could be misclassified as false positives.


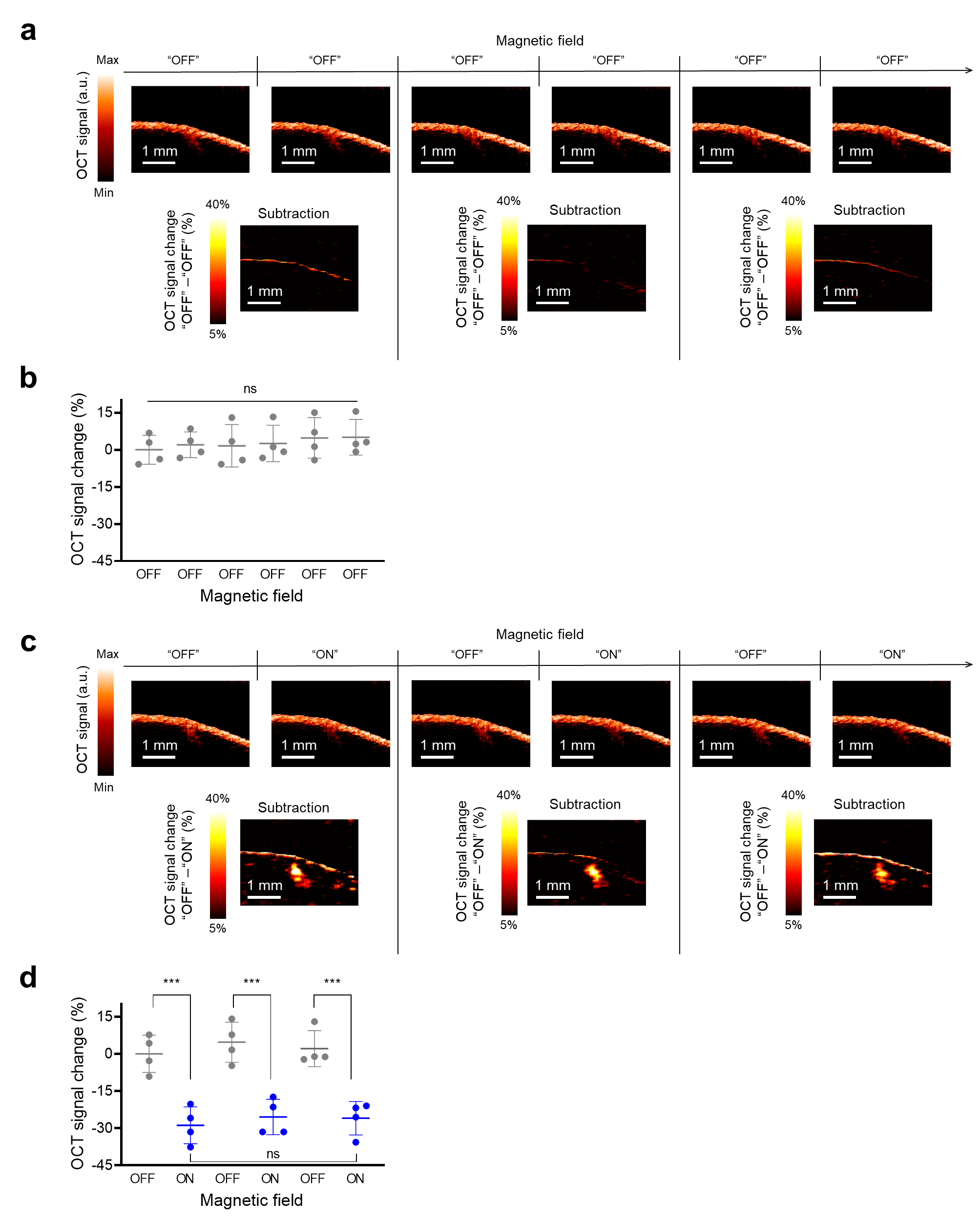


**Figure S33.** a-d) Reversible control of OCT signal generation from PMNAs (20 µg) in the mouse tissue after subcutaneous particle injection with the modulation of external magnetic field (n=4). Data are presented as the mean ± standard deviation. The statistical analyses for Figures S33b and S33d were conducted using a one-way ANOVA with Tukey post-hoc test. The statistically significant difference is represented as the asterisk (ns: non-significant, ***: p < 0.001). The imaging experiments were repeated independently four times and similar imaging results were obtained.
